# 6-Phosphogluconate dehydrogenase promotes mitochondrial fusion and immune suppression in tumor-associated monocytic suppressor cells

**DOI:** 10.1038/s41467-025-68102-8

**Published:** 2026-01-14

**Authors:** Saeed Daneshmandi, Qi Yan, Eduardo Cortes Gomez, Jee Eun Choi, Eriko Katsuta, Ehsan Gharib, Prashant K. Singh, Richard M. Higashi, Andrew N. Lane, Teresa W-M. Fan, Jianmin Wang, Elizabeth A. Repasky, Philip L. McCarthy, Hemn Mohammadpour

**Affiliations:** 1 Department of Cell Stress Biology, Roswell Park Comprehensive Cancer Center, Buffalo, NY USA; 2 Department of Immunology, Roswell Park Comprehensive Cancer Center, Buffalo, NY USA; 3 Department of Biostatistics & Bioinformatics, Roswell Park Comprehensive Cancer Center, Buffalo, NY USA; 4 Department of Hepatobiliary and Pancreatic Surgery, Graduate School of Medicine, Institute of Science Tokyo, Tokyo, Japan; 5 Department of Cancer Genetics & Genomics, Roswell Park Comprehensive Cancer Center, Buffalo, NY USA; 6 Department of Toxicology and Cancer Biology, Markey Cancer Center, Center for Environmental and Systems Biochemistry (CESB), Lexington, KY USA; 7 Department of Medicine, Roswell Park Comprehensive Cancer Center, Buffalo, NY USA

**Keywords:** Immunosuppression, Cancer immunotherapy, Tumour immunology, Monocytes and macrophages, Energy metabolism

## Abstract

The mechanisms underlying the metabolic adaptation of myeloid cells within the tumor microenvironment remain incompletely understood. Here, we identify 6-phosphogluconate dehydrogenase (6PGD), a rate-limiting enzyme in the pentose phosphate pathway (PPP), as an important regulator of monocytic-myeloid derived suppressor cell (M-MDSC) function. Our findings reveal that tumor M-MDSCs upregulate 6PGD expression via IL-6/STAT3 signaling. Blocking 6PGD, using either genetic or pharmacological approaches, impairs the immunosuppressive function of M-MDSCs and suppresses tumor growth. Mechanistically, 6PGD inhibition leads to the accumulation of its substrate, 6-phosphogluconate (6PG), within M-MDSCs, activates the JNK1-IRS1 and PI3K-AKT-pDRP1 signaling pathways, leading to mitochondrial fragmentation and elevated mitochondrial reactive oxygen species (ROS). This metabolic shift drives M-MDSCs toward an M1-like proinflammatory phenotype. Furthermore, 6PGD blockade synergizes with anti-PD-1 immunotherapy in a preclinical tumor model, substantially improving therapeutic outcomes. Our data reveals 6PGD as a possible therapeutic target to disrupt M-MDSC function and improve cancer immunotherapy outcomes.

## Abstract (web summary)

Myeloid derived suppressor cells (MDSC) use different metabolic mechanisms to adapt to the tumour microenvironment. Here the authors show that 6-phosphogluconate dehydrogenase (6PGD) is important for MSDC function and that blockade of 6PGD impaired MDSC function and suppresses tumour growth leading to metabolic and functional changes in the MDSC and a more pro-inflammatory phenotype.

## Introduction

The tumor microenvironment (TME) is a challenging metabolic landscape, characterized by a severe scarcity of nutrients, particularly glucose^1^. To adapt to this glucose imbalance, tumor cells undergo metabolic reprogramming to address their bioenergetic, biosynthetic, and redox demands for proliferation and growth^2^. Similarly, other cells within the TME, including diverse myeloid populations, must change their glucose metabolism to survive in these adverse conditions^3^. The TME is notably populated by various subsets of myeloid suppressor cells, including macrophages, monocytes, and neutrophils^4^. Among these, myeloid-derived suppressor cells (MDSCs) stand out as a distinct group, expanding in the TME and promoting tumor progression through potent immunosuppressive functions^5^. These MDSCs consist of two main populations: CD11b^+^Ly6C^+^Ly6G^-^F4/80^−^ monocytic-MDSC (M-MDSC) and CD11b^+^Ly6C^int^Ly6G^+^F4/80^−^ polymorphonuclear-MDSC (PMN-MDSC)^5,6^. We and others have shown that metabolic fitness, particularly OXPHOS capability, is crucial for driving the immunosuppressive function of MDSCs^7–9^. However, the mechanisms by which MDSCs maintain metabolic fitness in the TME remain unclear. In this study, we discovered that the tumor-infiltrating M-MDSCs overexpress 6-phosphogluconate dehydrogenase (6PGD), a rate-limiting enzyme in the pentose phosphate pathway (PPP), which plays a key role in regulating M-MDSC metabolic reprogramming, their suppressive functions and subsequent tumor outcomes.

The PPP is one of the key branches of glycolysis, also known as the hexose monophosphate shunt. The PPP consists of two distinct branches: the oxidative branch, which is largely irreversible, and the non-oxidative branch, which is reversible^10^. These pathways play critical roles in nucleotide biosynthesis, glutathione reduction, and NADPH regeneration^11^. Rate-controlling enzymes in the oxidative branch of the PPP, and glucose-6-phosphate dehydrogenase (G6PD), are regulated allosterically by their catalytic products and other metabolites^12^. The glucose flux through the PPP branches dynamically responds to metabolic stress, ensuring efficient glucose utilization^12^.

In this work, we found that 6PGD, but not other PPP enzymes, is significantly upregulated in tumor-associated MDSCs. This discovery prompted us to investigate the role of the PPP in MDSCs, focusing on the oxidative branch. We identified 6PGD as a metabolic checkpoint in the oxidative PPP of M-MDSCs that regulates immunosuppression in the TME. Our findings demonstrate that TME induces the oxidative branch of the PPP, upregulating 6PGD expression in M-MDSCs primarily through the STAT3 signaling pathway. Detailed mechanistic studies reveal that the accumulation of 6-phosphogluconate (6PG) triggers the phosphorylation of IRS-1^S307^. This subsequently enhances the PI3K-AKT-pDRP1 signaling pathway, leading to mitochondrial fission and enhanced production of mitochondrial reactive oxygen species (mROS). This metabolic reprogramming and mROS production alter M-MDSC function from immunosuppressive to immunostimulatory, resulting in the suppression of tumor growth in preclinical models. Furthermore, combining 6PGD blockade with anti-PD-1 immunotherapy significantly improves therapeutic efficacy in preclinical models. 6PGD, therefore, is a potential therapeutic target to disrupt M-MDSC function and enhance cancer immunotherapy.

## Results

### 6PGD in the oxidative branch of the PPP regulates M-MDSC immunosuppressive function

**Fig. 1:**
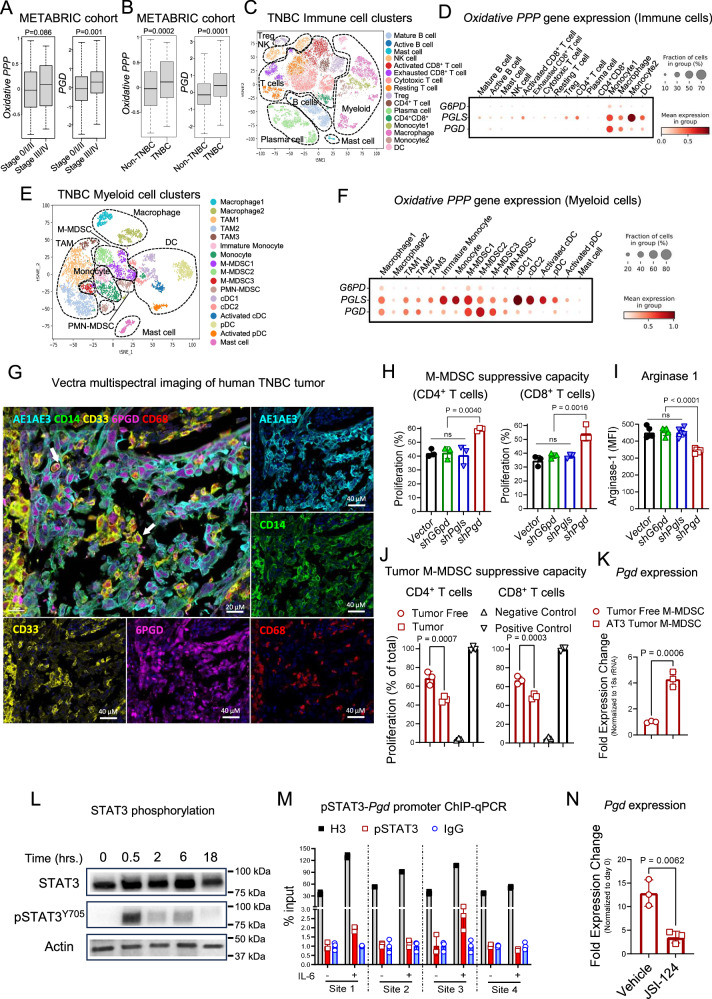
Identifying 6PGD as a metabolic vulnerability in human breast tumor myeloid suppressor cells regulated by tumor STAT3 signaling. Oxidative PPP and *PGD* gene expression were compared by stage (**A**) and by subtype, triple-negative breast cancer (TNBC) and non-TNBC patients (**B**) using the Molecular Taxonomy of Breast Cancer International Consortium (METABRIC) cohort dataset. *n* = 1903; Two-tailed *t* test. The tSNE plot (**C**) and PPP gene expression (**D**) of immune cell populations in primary human TNBC samples from the published single-cell RNA sequencing dataset (GSE161529). *n* = 8. The t-SNE plot of myeloid cell clusters (**E**) and oxidative PPP gene expression in myeloid cell compartments (**F**) of human TNBC samples. **G** Vectra multispectral imaging of human TNBC demonstrates expression of 6PGD (purple) in myeloid cells. Representative of one TNBC tumor. **H** Oxidative PPP checkpoints were blocked by shRNA in mice bone marrow (BM) derived MDSCs, then CD11b^+^Ly6C^+^Ly6G^−^ (M-MDSCs) were sorted. Their suppressive capacity was measured in co-culture with activated T cells at 1:8 (MDSC: T cell) ratio at 72 h. From two experiments with *n* = 3; One-way ANOVA. **I** The cells were generated as (**H**). Expression of arginase-1 was measured by flowcytometry. From two experiments with *n* = 4; One-way ANOVA. **J** CD11b^+^Ly6C^+^Ly6G^−^F4/80^−^ (M-MDSCs) were sorted from AT3 tumors and BM of tumor-free mice and co-cultured with activated T cells at 1:8 (MDSC: T cell) ratio at 72 h. Representative of two independent experiments, *n* = 3; One-way ANOVA. **K** M-MDSCs were prepared as in (**J**). *Pgd* expression was evaluated by Real-time PCR. Representative of two independent repeats. *n* = 3; Two-tailed *t* test. **L**, **M** Mouse BM cells were treated with IL-6 (40 ng/mL) and pSTAT3-Tyr705 phosphorylation was examined (**L**). pSTAT3 binding to *Pgd* promoter region was determined by ChIP-qPCR 30 minutes post IL-6 stimulation (**M**). Anti-histone H3 and IgG served as positive and negative controls, respectively. Representative of 3 independent experiments. **N** *Pgd* expression was evaluated by Real-time PCR on BM cells 48 h. post activation with IL-6 (40 ng/mL) with or without STAT3 inhibitor (JSI-124; 1 µM) treatment. Representative of 3 independent repeats. *n* = 3, two-tailed *t* test. All data are shown as mean ± SEM.

To understand the role of the PPP in tumors, we first explored the importance of the oxidative branch PPP in the TME of human breast cancer. We evaluated the PPP gene profile, including *PGD* (the gene encoding the 6PGD enzyme, the third enzyme in the PPP), in the publicly available Molecular Taxonomy of Breast Cancer International Consortium (METABRIC) cohort dataset (*n* = 1903)^13^. Expression of the PPP pathway and, in particular, *PGD* were elevated in patients with late-stage breast cancer (Fig. 1A) and in patients with more aggressive triple-negative breast cancer (TNBC) compared to non-TNBC (Fig. 1B). This suggests that the PPP plays a significant role in tumor progression.

To dissect the role of the PPP in different cells within the TME, we investigated the expression levels of key enzymes in the oxidative branch of the PPP (G6PD, PGLS, and 6PGD) in tumor-infiltrating immune cells (TIICs) using a previously published single-cell RNA sequencing (scRNA-seq) dataset of TNBC (GSE161529)^14^. Seurat analysis of the TIIC populations identified 16 immune cell clusters (Fig. 1C). Interestingly, PPP activity was particularly upregulated in myeloid cells, particularly at the 6PGD and PGLS checkpoints (Fig. 1D). Further analysis of TNBC myeloid subsets (Fig. 1E) showed that *PGD* checkpoint expression was specifically elevated in M-MDSC clusters, whereas *PGLS* expression was more broadly distributed, including dendritic cell (DC) subsets. This suggests that PGD expressions are more specific to M-MDSCs (Fig. 1F). To confirm the expression of 6PGD in human M-MDSCs, we examined the protein expression in a TNBC sample by Vectra multispectral imaging. The results demonstrated high 6PGD expression in tumor-infiltrating CD33^+^CD14^+^ M-MDSCs (Figs. 1G and S1). These findings highlight a key role for 6PGD in modulating the immunosuppressive function of tumor-infiltrating M-MDSCs. To examine if there is a similar role for PMN-MDSCs, we generated MDSCs in vitro from bone marrow (BM) of wild-type and 6PGD-deficient mice and isolated CD11b⁺Ly6C^int^Ly6G⁺ PMN-MDSCs via flow cytometry. We found that 6PGD inhibition had no significant impact on the suppressive function of PMN-MDSCs (Fig. S2). Thus, subsequent analyses focused exclusively on flow-sorted M-MDSCs.

To dissect the role of the PPP in M-MDSC functions, we examined the impact of all three key enzymes involved in the oxidative arm of the PPP. For this, each of the three enzymes was targeted in BM-derived M-MDSCs in vitro using short hairpin RNA (shRNA) (*shG6pd*, *shPgls*, and *shPgd*). Target knock-down efficacy through RNA interference (RNAi) was confirmed by western blot analysis (Fig. S3A–E). M-MDSC immunosuppressive effects on T cells were analyzed to assess M-MDSC functionality. T cell proliferation analyses showed that blocking 6PGD (*shPgd*), but not G6PD (*shG6pd*) or PGLS (*shPgls*), reduced the suppressive capacity of M-MDSCs when co-cultured with T cells (Fig. 1H). Furthermore, only 6PGD knock-down reduced the expression of arginase 1, a representative suppressive marker and a key enzyme involved in M-MDSC immunosuppression^15^ (Fig. 1I). These results underscore the critical role of 6PGD in regulating M-MDSC function within the TME.

Next, we explored the significance of 6PGD in M-MDSCs. We used AT3, a mouse breast tumor cell line derived from the primary mammary tumor cells of a transgenic middle T-antigen (MTAG) mouse^16^. M-MDSCs were isolated from AT3 tumor-bearing mice (Fig. S4), and their immunosuppressive function against T cell proliferation was compared with M-MDSCs isolated from the BM of tumor-free mice. As expected, tumor MDSCs exhibited potent suppressive capacity compared to control M-MDSCs (Fig. 1J). Additionally, 6PGD levels were significantly increased in tumor M-MDSCs, as confirmed by real-time PCR (Fig. 1K) and western blot (Fig. S1B, C), aligning with the scRNAseq data. These findings suggest a role for 6PGD in the immunosuppressive capacity of M-MDSCs. To further investigate the signaling pathway inducing 6PGD expression, we examined the role of STAT3, a well-documented transcription factor in M-MDSC development^17,18^. One of the major activators of STAT3 signaling in M-MDSCs is IL-6^17^, an abundant cytokine in the TME released by tumor cells, stromal cells such as fibroblasts, tumor-associated macrophages, and tumor-infiltrating M-MDSCs^19^. To examine the effect of IL-6 signaling on 6PGD expression during M-MDSC development, we first examined the early response time for IL-6 signaling activation on BM cells. As expected, IL-6 stimulation of BM cells resulted in STAT3 phosphorylation at Tyr705 30 min post-treatment (Fig. 1L). To determine whether this STAT3 activation led to 6PGD expression, we stimulated BM cells with IL-6 for 30 min and measured pSTAT3-Tyr705 binding to the *Pgd* promoter using chromatin immunoprecipitation–quantitative polymerase chain reaction (ChIP‒qPCR). pSTAT3 was found to bind two of the four potential binding sites in the *Pgd* promoter region (Figs. 1M and  S5A–C).

To confirm the role of STAT3 in *Pgd* regulation, we stimulated BM cells with IL-6 in the presence or absence of the STAT3 inhibitor JSI-124 (cucurbitacin I). Examination of *Pgd* expression by qPCR revealed that JSI-124 suppressed *Pgd* mRNA expression (Fig. 1N). These findings highlight the important role of 6PGD in MDSC development and demonstrate that *Pgd* expression is regulated by STAT3 signaling following IL-6 activation.

### Targeting the 6PGD metabolic checkpoint reduces M-MDSC-mediated T cell dysfunction and suppresses tumor growth

**Fig. 2:**
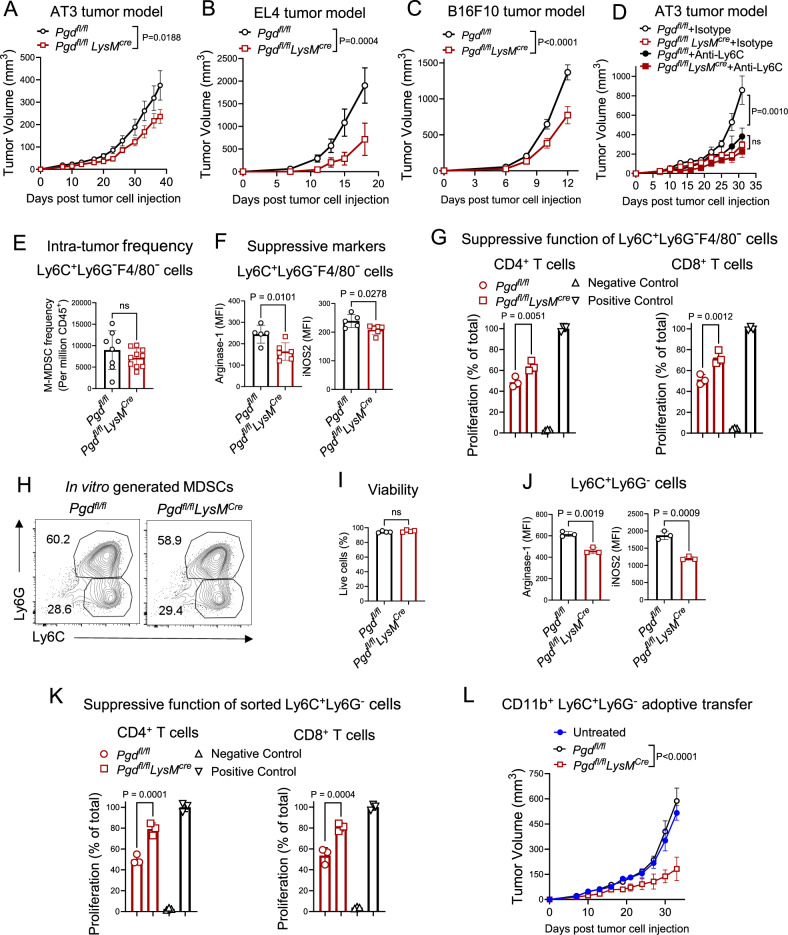
Blockade of 6PGD overcomes M-MDSC-mediated T cell dysfunction and suppresses tumor growth of preclinical models. *Pgd*^*fl/fl*^ and *Pgd*^*fl/fl*^*LysM*^*Cre*^ mice were inoculated subcutaneously (SC) with 5 × 10^5^ AT3 breast cancer (**A**), 1 × 10^6^ EL-4 lymphoma (**B**), or 2 × 10^5^ B16F10 melanoma (**C**) and tumor growth was monitored. *n* = 10 mice per group; Two-way ANOVA. **D** *Pgd*^*fl/fl*^ and *Pgd*^*fl/fl*^*LysM*^*Cre*^ mice inoculated SC with 5 × 10^5^ AT3 cells monitored for tumor growth after 200 μg anti-Ly6C depleting monoclonal antibody (mab) or isotype control (twice weekly; i.p.) administration. *n* = 5 mice per group; Two-way ANOVA. Intra-tumor frequency (**E**), and immunosuppressive marker expression of Arginase 1 (Arg 1) and inducible Nitric Oxide synthase 2 (iNOS2) (**F**) were evaluated on AT3 tumor infiltrating CD11b^+^Ly6C^+^Ly6G^−^F4/80^−^ (M-MDSC) cells. *n* = 10 mice per group, Two-tailed *t* test. **G** M-MDSC cells sorted from AT3 tumor bearing *Pgd*^*fl/fl*^ and *Pgd*^*fl/fl*^*LysM*^*Cre*^ mice were co-cultured with anti-CD3/anti-CD28 mAb-activated T cells at 1:8 (MDSC: T cell) ratio. T cell proliferation was evaluated at 72 h. Unsimulated T cells and T cells stimulated with anti-CD3 and anti-CD28 served as controls. Representative of two independent experiments with *n* = 3; Two-way ANOVA. **H**, **I** MDSCs were generated in vitro using IL-6 (40 ng/mL) and GM-CSF (40 ng/mL) for 4 days from the BM of *Pgd*^*fl/fl*^ and *Pgd*^*fl/fl*^*LysM*^*Cre*^ mice (**H**). M-MDSC viability was measured by Annexin V staining (**I**). Representative of three independent experiments with *n* = 4, Two-tailed *t* test. **J** M-MDSCs were generated as in (**H**, **I**). Arginase 1 and iNOS2 expressions were evaluated flow cytometry. Representative of three independent experiments with *n* = 3; Two-tailed *t* test. **K** In vitro generated M-MDSC as in (**H**, **I**) were co-cultured with anti-CD3/anti-CD28 mab activated T cells at 1:8 (MDSC: T cell) ratio and T cell proliferation was evaluated at 72 h. Representative of three independent experiments with *n* = 3; One-way ANOVA. **L** To examine M-MDSCs suppressive function in vivo, 5 × 10^5^ AT3 tumor cells were injected SC and sorted CD11b^+^Ly6C^+^Ly6G^−^ M-MDSC (1 × 10^6^ cells) were intravenously injected on days 3 and 6 and tumor growth was monitored. Untreated controls received AT3 cells only. *n* = 6 per group; Two-way ANOVA. All data are shown as mean ± SEM.

We have so far shown that 6PGD is a key PPP enzyme induced in M-MDSCs after STAT3 activation and plays a critical role in regulating M-MDSC function in vitro. We asked if targeting 6PGD in vivo impacts tumor growth. We generated a *Pgd* conditional knockout in myeloid cell lineage mice with a flox-targeted *Pgd* allele (*Pgd*^*fl/fl*^). These mice were crossed with *LysM*^*Cre*^ mice to generate *Pgd*^*fl/fl*^*LysM*^*Cre*^ mice to assess the role of 6PGD in modulating M-MDSC function. In this model, 6PGD is inactivated in MDSCs upon Cre enzyme expression. To determine whether 6PGD-deficient MDSCs can control tumor growth, we injected *Pgd*^*fl/fl*^*LysM*^*Cre*^ and *Pgd*^*fl/fl*^ mice with AT3 breast cancer (Fig. 2A), EL-4 lymphoma (Fig. 2B), or B16F10 melanoma (Fig. 2C) cell lines. Tumor growth showed a significant decrease in *Pgd*^*fl/fl*^*LysM*^*Cre*^ mice compared to *Pgd*^*fl/fl*^ control mice in all tumor models.

Lysozyme, (the base gene used to generate *LysM*^*Cre*^ mice) is also expressed in macrophages and some dendritic cells^20^. To specifically evaluate the role of 6PGD in M-MDSCs we targeted Ly6C which is highly expressed on M-MDSCs (Ly6C^hi^)^21^. MDSCs were depleted using anti-Ly6C monoclonal antibodies (mAbs) in *Pgd*^*fl/fl*^ and *Pgd*^*fl/fl*^*LysM*^*Cre*^ AT-3 tumor-bearing mice. We previously confirmed the efficacy of anti-Ly6C mab to deplete M-MDSCs in vivo^22^. Depletion of M-MDSCs in *Pgd*^*fl/fl*^ AT3 tumor-bearing mice resulted in a tumor growth rate similar to that in *Pgd*^*fl/fl*^*LysM*^*Cre*^ mice. This suggests that 6PGD deletion in M-MDSCs is a major driver of tumor control observed in *Pgd*^*fl/fl*^*LysM*^*Cre*^ mice (Fig. 2D).

We next explored the phenotypic and functional properties of M-MDSCs in the *Pgd*^*fl/fl*^*LysM*^*Cre*^ and *Pgd*^*fl/fl*^ AT3 tumor models. Flowcytometry analysis showed no significant difference in the frequency of intra-tumoral M-MDSCs (Fig. 2E). Examination of infiltrating M-MDSCs showed reduced expression of arginase 1 and inducible nitric oxide synthase 2 (iNOS2) (immunosuppressive markers) in *Pgd*^*fl/fl*^*LysM*^*Cre*^ compared to *Pgd*^*fl/fl*^ AT3 tumor models (Fig. 2F). In functional assays, co-culture of sorted M-MDSCs from AT3 tumor-bearing mice with anti-CD3/anti-CD28 activated T cells resulted in a lower suppressive effect of *Pgd*^*fl/fl*^*LysM*^*Cre*^ M-MDSCs compared to *Pgd*^*fl/fl*^ M-MDSCs on both CD4^+^ and CD8^+^ T cells (Fig. 2G).

To confirm the role of 6PGD blockade to reduce the M-MDSC suppressive phenotype observed in vivo, MDSCs were generated in vitro from the BM of *Pgd*^*fl/fl*^*LysM*^*Cre*^ and *Pgd*^*fl/fl*^ mice (Fig. 2H). We first checked the potential cytotoxic effect of 6PGD deletion and found no significant effect on M-MDSC viability, as measured by Annexin V staining (Fig. 2I). Flow cytometry analysis of differentiated cells showed lower expression of Arginase 1 and iNOS2 immunosuppressive markers on M-MDSC (Fig. 2J) cells in *Pgd*^*fl/fl*^*LysM*^*Cre*^ compared to the *Pgd*^*fl/fl*^ group. In functional assays, in vitro-generated M-MDSCs were co-cultured with activated T cells. Analysis of T cell proliferation indicated that *Pgd*^*fl/fl*^*LysM*^*Cre*^ M-MDSCs demonstrated reduced suppressive capacity compared to *Pgd*^*fl/fl*^ M-MDSCs on both CD4^+^ and CD8^+^ T cells (Fig. 2K).

In further support of these findings, we examined the immunoregulatory function of in vitro-generated *Pgd*^*fl/fl*^*LysM*^*Cre*^ and *Pgd*^*fl/fl*^ M-MDSCs in in vivo tumor models using adoptive transfer. AT3 tumors were established by SC injection, followed by intravenous transfer of in vitro-generated M-MDSCs on days 3 and 6. Lower tumor growth was observed in mice receiving *Pgd*^*fl/fl*^*LysM*^*Cre*^ M-MDSCs compared to the *Pgd*^*fl/fl*^ group (Fig. 2L).

These studies confirm that 6PGD expression in tumor-infiltrating M-MDSCs is essential for maintaining their immunosuppressive function and contributes significantly to tumor progression.

### 6PGD deficiency induces a unique transcriptomics profile with immunostimulatory signatures in M-MDSCs

**Fig. 3:**
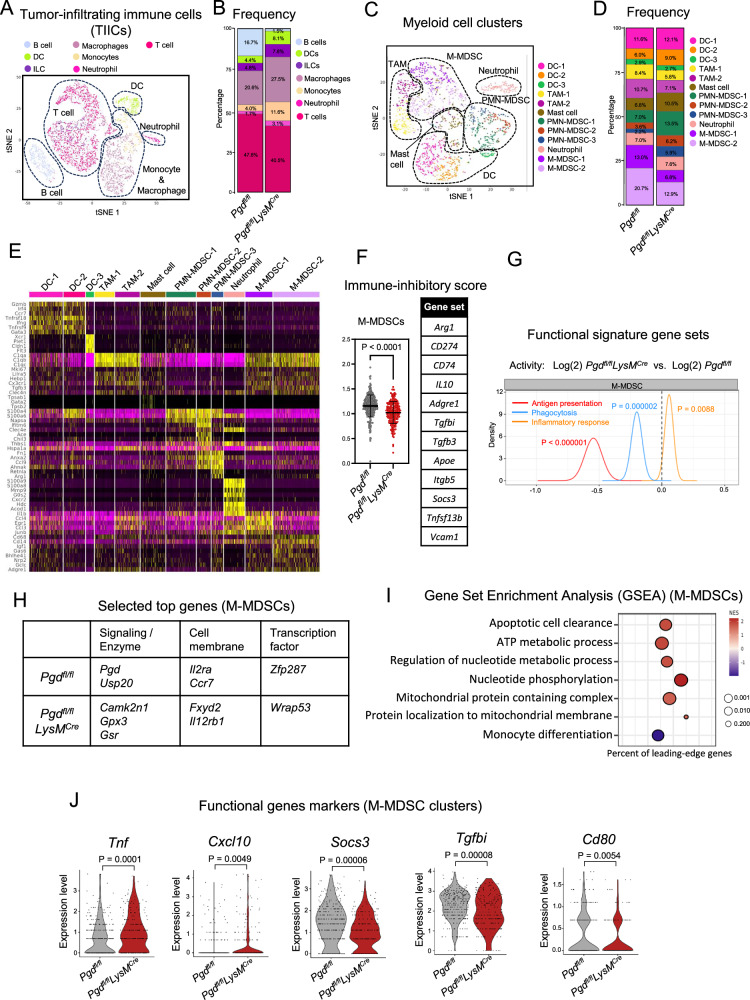
6PGD blockade during myeloid suppressor cell differentiation induces transformational transcriptomics profile in M-MDSC subpopulations. **A**, **B** *Pgd*^*fl/fl*^ and *Pgd*^*fl/fl*^*LysM*^*Cre*^ mice were inoculated subcutaneously (SC) with AT3 breast cancer cells (5 × 10^5^ cells per mouse). The t-SNE visualization of the tumor infiltrating immune cell transcriptomics profile evaluated by single cell RNA sequencing (scRNA-seq) after 30 days are shown in (**A**). The frequency of each cluster in *Pgd*^*fl/fl*^*LysM*^*Cre*^ and *Pgd*^*fl/fl*^ tumor bearing mice is shown in (**B**). **C**, **D** t-SNE visualization of AT3 tumor-infiltrating myeloid cells from *Pgd*^*fl/fl*^*LysM*^*Cre*^ and *Pgd*^*fl/fl*^ mice are shown in (**C**). The myeloid cell frequencies from *Pgd*^*fl/fl*^*LysM*^*Cre*^ and *Pgd*^*fl/fl*^ mice are shown in (**D**). **E** The Heatmap displays the scaled expression of top gene signatures in myeloid cell clusters from (**C**); The gene expression is coded with a scale based on the *z* score distribution from lowest expression: −2 (purple) to highest expression: 2 (yellow). **F** M-MDSC immune-inhibitory scores of *Pgd*^*fl/fl*^*LysM*^*Cre*^ and *Pgd*^*fl/fl*^ AT3 tumor-bearing mice are shown. The gene set used for the immune-inhibitory score is listed in the table in (**F**). Two-tailed *t* test. **G** Comparison of scores of antigen presentation, phagocytosis and inflammatory response of M-MDSCs between *Pgd*^*fl/fl*^*LysM*^*Cre*^ and *Pgd*^*fl/fl*^ AT3 tumor-bearing mice using QuSAGE. **H** The highest gene expression of signaling/enzyme proteins, cell membrane markers, and transcription factors differed significantly between M-MDSCs from *Pgd*^*fl/fl*^ and *Pgd*^*fl/fl*^*LysM*^*Cre*^ tumor-bearing recipients. Genes are ranked by *p* value. *P*  <  0.05 indicated statistical significance. **I** A selected gene set enrichment analysis (GSEA) based on the Gene Ontology (GO) database shows the M-MDSC ratio of *Pgd*^*fl/fl*^*LysM*^*Cre*^ to *Pgd*^*fl/fl*^ AT3 tumor-bearing mice. The percentage of genes showing an increase or decrease in expression is presented as the percentage of leading-edge genes. The circle range represents the significance threshold. Red: High expression; Blue: Low expression. The gene expression is coded from lowest expression: −2 (blue) to highest expression: 2 (red). NES normalized enrichment score. **J** The functional marker gene expression levels on M-MDSC clusters in *Pgd*^*fl/fl*^*LysM*^*Cre*^ to *Pgd*^*fl/fl*^ AT3 tumor-bearing mice. Two-tailed *t* test.

Our in vitro and in vivo experiments indicated that 6PGD deficiency causes a significant decrease in the immunosuppressive function of M-MDSCs. To dissect the immunophenotype of myeloid cell compartments in the TME, transcriptomic profiles of TIICs (CD45^+^ cells) were evaluated in AT3 tumors from *Pgd*^*fl/fl*^*LysM*^*Cre*^ and *Pgd*^*fl/fl*^ tumor-bearing mice using scRNA-seq 30 days post-tumor inoculation. Visualization of TIIC clusters in a t-SNE plot identified distinct clusters of T cells, B cells, innate lymphoid cells (ILCs), and myeloid cells (macrophages, monocytes, neutrophils and dendritic cells (DCs) (Fig. 3A). The immune cell proportions were calculated in the two mice models (Fig. 3B). To further characterize the myeloid compartments, we clustered myeloid cells into 12 subpopulations, including 2 subsets of tumor associated macrophages (TAMs), 3 subsets of DCs, 1 subset of mast cells, 1 subset of neutrophils, 3 subsets of PMN-MDSCs, and 2 subsets of M-MDSCs (Fig. 3C, D). The clusters were defined based on the top differentially expressed genes correlated with each subpopulation, summarized in the heatmap (Fig. 3E).

Examination of the gene signatures confirmed that M-MDSCs exhibited reduced immune-inhibitory scores^23^ in the TIICs of *Pgd*^*fl/fl*^*LysM*^*Cre*^ compared to those in *Pgd*^*fl/fl*^ tumor-bearing mice (Fig. 3F). Furthermore, the M-MDSC subpopulations demonstrated lower antigen presentation and phagocytosis scores^23^ while displaying higher inflammatory responses in *Pgd*^*fl/fl*^*LysM*^*Cre*^ mice compared to *Pgd*^*fl/fl*^ mice (Fig. 3G). This suggests that 6PGD deficiency drives M-MDSCs in the TME toward a more M1-like proinflammatory phenotype.

The expression levels of genes encoding signaling/enzyme proteins, cell membrane markers, and transcription factors were compared between TIICs of *Pgd*^*fl/fl*^*LysM*^*Cre*^ mice and *Pgd*^*fl/fl*^ AT3 tumor-bearing mice (Fig. 3H). As expected, *Pgd* expression was one of the top genes highly expressed in M-MDSC subsets of *Pgd*^*fl/fl*^ mice compared to *Pgd*^*fl/fl*^*LysM*^*Cre*^ mice.

Among highly expressed genes, a comparative analysis revealed that genes associated with the protumor functions (*USP20, Il2ra, and Ccr7*)^24–26^ were upregulated in *Pgd*^*fl/fl*^ M-MDSCs (Fig. 3H). In contrast, genes related to metabolic fitness (*Gpx3, Gsr, Camk2n1*)^27,28^, antitumor function (*Fxyd2, Il12rb1*)^29,30^, and survival (*Wrap53*)^31^ were upregulated in *Pgd*-deficient M-MDSCs (Fig. 3H). Gene Set Enrichment Analysis (GSEA) using Gene Ontology (GO) terms was performed to compare the M-MDSC signature profiles of *Pgd*^*fl/fl*^*LysM*^*Cre*^ and *Pgd*^*fl/fl*^ AT3 tumor-bearing mice (Fig. 3I). The analysis revealed that gene sets promoting monocyte differentiation were downregulated in *Pgd*-deficient M-MDSCs, whereas gene sets related to apoptotic cell clearance were enriched (Fig. 3I). Additionally, *Pgd*-deficient M-MDSCs showed enrichment in gene sets related to nucleotide metabolism, nucleotide phosphorylation, and ATP generation, consistent with enhanced proinflammatory responses (Fig. 3I). Furthermore, these cells showed enrichment in mitochondrial protein complexes and protein localization to the mitochondrial membrane (Fig. 3I). We further analyzed the expression of key genes related to M-MDSC phenotype and function. *Pgd*-deficient M-MDSCs expressed elevated levels of the proinflammatory marker *Tnf* and *Cxcl10* alongside reduced levels of the suppressive markers *Socs3* and *Tgfbi* (Fig. 3J). The lower expression of the costimulatory marker *Cd80* corresponded to the reduced antigen presentation capacity of *Pgd*-deficient M-MDSCs (Fig. 3G, J).

These findings indicate that inhibiting the 6PGD function shifts the gene expression profile and phenotype of tumor M-MDSCs toward a proinflammatory state.

### 6PGD deficiency in M-MDSCs induces metabolic reprogramming marked by higher glycolysis levels and decreased oxidative phosphorylation

**Fig. 4:**
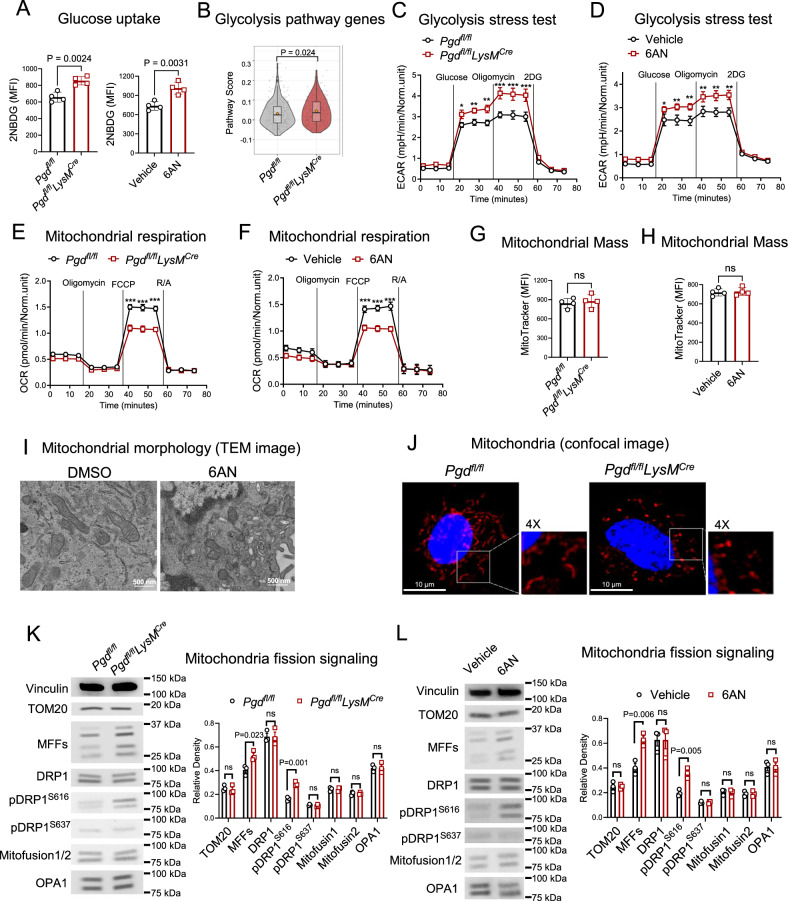
6PGD blockade induces metabolic reprograming marked by higher glycolysis and higher mitochondrial fission in M-MDSCs. **A** MDSCs were generated in vitro using IL-6 (40 ng/mL) and GM-CSF (40 ng/mL) from bone marrow (BM) of *Pgd*^*fl/fl*^ and *Pgd*^*fl/fl*^*LysM*^*Cre*^ mice. In parallel experiments, MDSCs were generated in the presence of 6-aminonicotinamide (6AN) (5 µM) or vehicle (DMSO). Glucose uptake by M-MDSCs was measured by 2-(N-(7-nitrobenz-2-oxa-1,3-diazol-4-yl) amino)-2-deoxyglucose (2-NBDG) in *Pgd*^*fl/fl*^*LysM*^*Cre*^ and 6AN compared to their controls in flowcytometry. *n* = 4; Two-tailed t-test. **B** Expression of glycolytic pathway genes was analyzed on M-MDSCs of AT3 tumor bearing *Pgd*^*fl/fl*^*LysM*^*Cre*^ versus *Pgd*^*fl/fl*^ mice (M-MDSC clusters in Fig. 3 (**C**). Two-tailed t*-*test. Glycolytic pathway in sorted M-MDSC generated as in A was measured in *Pgd*^*fl/fl*^*LysM*^*Cre*^ compared to *Pgd*^*fl/fl*^ (**C**) and 6AN treated compared to vehicle (**D**). ^∗^*p* < 0.05; ^∗∗^*p* < 0.01, ^∗∗∗^*p* < 0.001; *n* = 3; Two-tailed t-test. **E**, **F** Sorted M-MDSC generated as in (**A**) underwent Seahorse Mito Stress metabolic flux assay. A lower rate of mitochondrial respiration (lower oxygen consumption rate (OCR)) was seen in *Pgd*^*fl/fl*^*LysM*^*Cre*^ compared to *Pgd*^*fl/fl*^ M-MDSCs (**E**) and 6AN-treated compared to vehicle (**F**) MDSCs. ^∗∗∗^*p* < 0.001; *n* = 3; Two-tailed t-test. **G**, **H** Sorted M-MDSCs generated as per A were assessed for mitochondrial mass by MitoTracker DeepRed staining. *n* = 4; Two-tailed t-test. **I** MDSCs generated and treated with 6AN or vehicle as per **A** were examined for mitochondrial morphology by Transmission electron microscopy (TEM). There was increased mitochondrial fragmentation consistent with the mitochondrial fission phenotype with 6AN treatment. The result is a representative of TME imaging. **J** M-MDSCs generated as per **A** were stained with MitoTracker Deep Red and examined by confocal microscopy for mitochondrial morphology. Nucleus is stained with DAPI (blue). The mitochondria are shown in red. Nuclei are stained blue, and mitochondria are red. original magnification = ×20. Result is a representative of confocal imaging. **K**, **L** Mitochondrial fission/fusion status was evaluated in M-MDSCs (generated as per (**A**)) by Mitochondrial Dynamics Antibody Sampler Kit in western blot analysis. Blots were processed in parallel to samples derived from the same experiment. *n* = 3; Two-tailed t-test. All data are shown as mean ± SEM.

The oxidative branch of PPP is a key route for glucose consumption in central carbon metabolism, branching off from G6P in the early steps of glycolysis^11^. We investigated how 6PGD function affects glucose consumption and central carbon metabolism. MDSCs were generated in vitro from the BM of both *Pgd*^*fl/fl*^ and *Pgd*^*fl/fl*^*LysM*^*Cre*^ mice. We also pharmacologically inhibited 6PGD in BM-derived MDSCs using 6-Aminonicotinamide (6AN) (5 µM) or a vehicle control. Glucose uptake, measured by the 2-(N-(7-nitrobenz-2-oxa-1,3-diazol-4-yl)amino)−2-deoxyglucose (2-NBDG) fluorescent tracer, showed an increased glucose uptake in *Pgd*-deficient M-MDSCs and 6AN treated M-MDSCs (Fig. 4A). These findings were consistent with scRNA-seq data, which revealed an enrichment in the glycolytic pathway genes in *Pgd*-deficient tumor M-MDSC clusters (Fig. 4B). Seahorse glycolysis stress tests revealed that *Pgd*-deficient M-MDSCs (Fig. 4C) displayed elevated glycolytic activity, as indicated by increased extracellular acidification rates (ECAR) compared to controls. This finding was validated by selective 6PGD inhibition by 6AN. 6AN-treated M-MDSCs (Fig. 4D) similarly exhibited enhanced glycolysis, confirming that 6PGD inhibition shifts M-MDSC metabolism toward a glycolytic phenotype.

Glycolysis is linked to pro-inflammatory responses in myeloid cells^32^, while mitochondrial respiration is associated with immunosuppressive responses^7,8^. To determine the impact of *Pgd* deficiency on mitochondrial respiration, we performed Mito-stress Seahorse analysis on WT and *Pgd-*deficient M-MDSCs. This analysis revealed significantly reduced mitochondrial respiration in both *Pgd*-deficient (Fig. 4E) and 6AN-treated M-MDSCs (Fig. 4F), compared to controls. The flux seahorse analysis findings demonstrate that PPP inhibition at the 6PGD checkpoint enhances glycolysis while suppressing mitochondrial respiration in M-MDSCs.

Mitochondrial dynamics are critical for metabolic reprogramming^33,34^. Since *Pgd* deficiency induced this reprogramming, we examined mitochondrial structure and function in *Pgd*-deficient M-MDSCs. Mitochondrial structural changes can denote metabolic modulation. This includes fusion of two mitochondria and fission into two^34^. Our scRNA-seq data showed significant enrichment in genes associated with mitochondrial membrane rearrangements in *Pgd*-deficient M-MDSCs (Fig. 3I). MitoTracker Deep Red FM staining revealed no difference in mitochondrial mass between *Pgd*^*fl/fl*^*LysM*^*Cre*^ and *Pgd*^*fl/fl*^ M-MDSCs or 6AN or DMSO-treated M-MDSCs (Fig. 4G, H). Electron microscopy showed increased mitochondrial fragmentation in 6AN-treated MDSCs, consistent with fission morphology (Fig. 4I). Using confocal microscopy we found smaller, more circular mitochondria in *Pgd*^*fl/fl*^*LysM*^*Cre*^ compared to *Pgd*^*fl/fl*^ M-MDSCs (Fig. 4J), consistent with the electron microscopy findings. To validate these observations, we analyzed mitochondrial fission and fusion markers using the Mitochondrial Dynamics Antibody Sampler Kit in western blot analysis. Tom20, a mitochondrial mass marker, localized in the outer mitochondrial membrane, remained unchanged by 6PGD blockade (Fig. 4K, L), corroborating the MitoTracker results. Mitochondrial fission factor (MFF) and phosphorylated Dynamin-related protein 1 (DRP1) at Ser616 (pDRP1^S616^), regulators of mitochondrial fission, increased in *Pgd*-deficient M-MDSCs (Fig. 4K) and 6AN-treated M-MDSCs (Fig. 4L). In contrast, levels of mitofusin1 and 2 (dynamin-like GTPases) and Optic atrophy 1 (OPA1), all of which facilitate mitochondrial fusion, were unchanged (Fig. 4K, L).

These results confirm that 6PGD blockade in M-MDSCs induces a significant metabolic shift leading to significant structural changes in mitochondria.

### The accumulation of the metabolite 6-phosphogluconate (6PG) induces phosphorylation of IRS-1 at Ser307, enhancing M-MDSC glycolysis

To explore the mechanistic basis for these metabolic and functional changes, we investigated PPP glucose consumption. We measured glucose utilization in the PPP using stable isotope-resolved metabolomics (SIRM) analysis. WT and *Pgd*-deficient MDSCs were generated in vitro and treated with D_7_-glucose. Metabolite patterns were analyzed using Ion Chromatography-Ultra High-Resolution Mass Spectrometry (IC-UHRMS) at the 8-hour timepoint. The SIRM data revealed an effective 6PGD blockade and a significant accumulation of deuterated 6PG upon 6PGD inhibition in both *Pgd-*deficient (Fig. S6A-b) and 6AN-treated MDSCs (Fig. S6B-b). Ribose-5-Phosphate (R5P), the product of 6PGD, also accumulated (Figs. S6A-c and S6B-c), indicating the activation of the non-oxidative branch to compensate for the loss of 6PGD function. Enhanced non-oxidative PPP activity was consistent with increased levels of D-labeled S7P, E4P, F6P, and G3P (Figs. S6A-d-g and S6B-d-g).

**Fig. 5:**
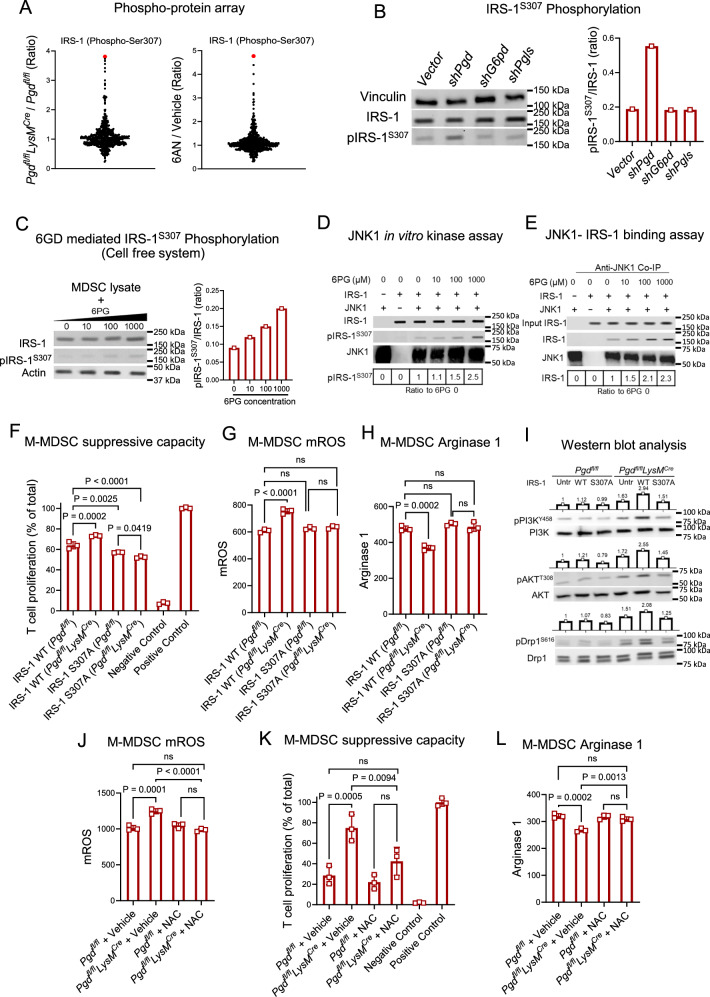
6-phosphogluconate (6PG) is a functional metabolite, directing M-MDSC suppression by inducing IRS-1^S307^ phosphorylation and IRS1-PI3K-AKT signaling. **A** MDSCs were generated in vitro using IL-6 (40 ng/mL) and GM-CSF (40 ng/mL) from bone marrow (BM) of *Pgd*^*fl/fl*^ and *Pgd*^*fl/fl*^*LysM*^*Cre*^ mice or wild type (WT) mice with 6AN (5 µM) or vehicle. Sorted MDSCs on day 4 were analyzed for 1318 signaling target proteins by Phospho-Explorer Antibody Array (Full moon BioSystem). **B** Oxidative PPP checkpoints were blocked by shRNA in WT BM-derived MDSCs. Phospho-IRS-1^S307^ levels were examined on sorted M-MDSCs. Blots were processed parallel to samples from the same experiment. Results are representative of two repeats. **C** 6PG (10, 100, 1000 µM) was added to MDSC cell free lysates for 30 min and IRS-1^S307^ phosphorylation measured by western blot. Blots were processed parallel to samples from the same experiment. Results are representative of two repeats. **D**, **E** Immunoprecipitated JNK1 and IRS-1 (substrate) were incubated with increasing 6PG concentrations (10, 100, 1000 µM) in an in vitro kinase assay (**D**). In the same experiment, JNK1-IRS-1 binding was evaluated by co-immunoprecipitation (Co-IP) using anti-JNK1 (**E**). Blots were processed parallel to samples from the same experiment. Results are representative of two repeats. **F** Serine to Alanine (S307A) site-specific mutation in IRS-1 was conducted in M-MDSCs (generated as in (**A**)). Flowcytometry-sorted M-MDSCs were co-cultured with anti-CD3/anti-CD28 activated T cells (CFSE-labeled) at a 1:8 (M-MDSC: T cell) ratio. T cell proliferation was evaluated at 72 h. *n* = 3; One-way ANOVA. **G**, **H** M-MDSCs were generated as in (**A**, **F**). Mitochondrial ROS (mROS) production (**G**) and Arginase 1 expression (**H**) was examined on M-MDSC by flow cytometry. *n* = 3; One-way ANOVA. **I** Sorted M-MDSCs were prepared as in (**A**, **F**). Levels of PI3K (top), AKT (middle) and Drp1^S616^ (bottom) phosphorylation were determined by western blot analysis. Blots were processed parallel to samples from the same experiment. Result is representative of two repeats. **J**–**L** MDSCs were generated as in (**A**, **F**), with N-Acetylcysteine (NAC: 0.5 mM) or vehicle (PBS). mROS production in M-MDSCs (**J**), suppressive capacity in co-culture with T cells (**K**) and Arginase expression levels (**L**) determined. *n* = 3; One-way ANOVA. All data are shown as mean ± SEM.

To understand the intracellular mechanisms of metabolic reprogramming observed in 6PGD-deficient M-MDSCs, we investigated key signaling pathways and molecular regulators associated with glycolytic activation, mitochondrial function, and immunosuppressive activity. We used a phosphoproteomic array, screening 1318 signaling proteins using a Phospho Explorer Antibody Array (Full Moon BioSystem) in *Pgd*-deficient and 6AN-treated MDSCs (Fig. 5A). The most significantly altered target in both MDSC types was phospho-insulin receptor substrate-1 (pIRS-1) at Ser307 (pIRS-1^S307^; (equivalent to human Ser312) (Fig. 5A). IRS-1 is part of the IRS family, which is phosphorylated in response to insulin, IGF-1, and cytokines^35^. While in vitro studies suggest that IRS-1^S307^ promotes insulin resistance, knock-in studies in mice suggest that Ser307 positively regulates insulin signaling^36^.

Based on the screening studies, we hypothesized that 6PG accumulation might drive IRS-1^S307^ phosphorylation in M-MDSCs. To test this, we inhibited key enzymes in the oxidative PPP (*G6pd*, *Pgls*, and *Pgd*) using shRNA in BM-derived M-MDSCs. Only the *Pgd* knockdown resulted in increased pIRS-1^S307^ levels (Fig. 5B). Given the significant accumulation of 6PG in both *Pgd-deficient* and 6AN-treated cells (Figs. S6A-b, S6B-b), we investigated whether 6PG could induce IRS-1^S307^ phosphorylation. Indeed, adding increasing concentrations of 6PG to cell-free lysates enhanced IRS-1^S307^ phosphorylation (Fig. 5C).

IRS-1^S307^ phosphorylation is mediated by the c-Jun N-terminal kinase 1 (JNK1)^37^. To further explore if 6PG regulates the JNK1-IRS1 interaction, we conducted an in vitro kinase assay using purified JNK1 and IRS-1. The results showed that increasing 6PG concentrations promoted JNK1 phosphorylation of IRS-1^S307^ (Fig. 5D). Increased 6PG levels also increased JNK1-IRS-1 binding (Fig. 5E). These findings confirm that the 6PG metabolite induces JNK1-IRS1 binding and phosphorylation of IRS-1^S307^.

To assess the functional impact of 6PG-mediated IRS-1^S307^ phosphorylation on M-MDSCs, we generated M-MDSCs with a site-directed mutation at IRS-1^S307^, substituting serine for alanine (IRS-1^S307A^) (Fig. S7). The mutation abrogated the decrease in suppressive capacity observed in 6PGD-blocked, wild-type IRS-1 (IRS-1^S307^) transfected M-MDSCs (Fig. 5F). Flow cytometry analysis confirmed that IRS-1^S307A^ mutation downregulated mROS production in 6PGD-deficient M-MDSCs (Fig. 5G). In addition, the expression of arginase 1 was downregulated by the IRS-1^S307A^ mutation in 6PGD-deficient M-MDSCs (Fig. 5H).

We examined mitochondrial fission, a key component of 6PGD blockade-induced metabolic reprogramming (previously described in Fig. 4). The IRS-1^S307A^ mutation reduced Drp1^S616^ phosphorylation and downstream of JNK1-IRS-1 signaling as AKT and PI3K phosphorylation (Fig. 5I). Confocal microscopy further confirmed that the IRS-1^S307A^ mutation prevented the formation of mitochondrial fission structures in 6PGD-deficient M-MDSCs (Fig. S8).

One of the outcomes from changes in mitochondrial structure is a shift in mROS production, serving as signaling for the induction of a proinflammatory phenotype. The MitoSox staining confirmed that 6PGD blockade induces the generation of mROS in M-MDSCs, which can be prevented by N-acetylcysteine (NAC) antioxidant treatment (Fig. 5J). In the in vitro suppression assay, NAC treatment abrogates the T cell stimulatory capacity of 6PGD-deficient M-MDSCs (Fig. 5K). In addition, NAC treatment elevated arginase 1 expression of 6PGD-deficient M-MDSCs (Fig. 5L). These findings demonstrate that inhibiting 6PGD in M-MDSCs results in the accumulation of 6PG, which promotes interactions between JNK1 and IRS-1, leading to increased IRS-1S307 phosphorylation. This process enhances glucose uptake and its utilization through glycolysis while simultaneously reducing mitochondrial reliance by inducing mitochondrial fission. These changes in mitochondrial structure induce mROS generation, which serves as a signal to induce proinflammatory responses in 6PGD-deficent M-MDSCs.

Significantly, this research highlights a key physiological role of the PPP in M-MDSCs. It reveals that 6PG accumulation disrupts the metabolic fitness of M-MDSCs. To counteract this disruption, M-MDSCs overexpress 6PGD as a regulatory mechanism to prevent excessive cellular 6PG buildup and to maintain metabolic stability.

### 6PGD inhibition reduces MDSC immunosuppression and enhances the efficacy of anti-PD-1 immune checkpoint blockade

**Fig. 6:**
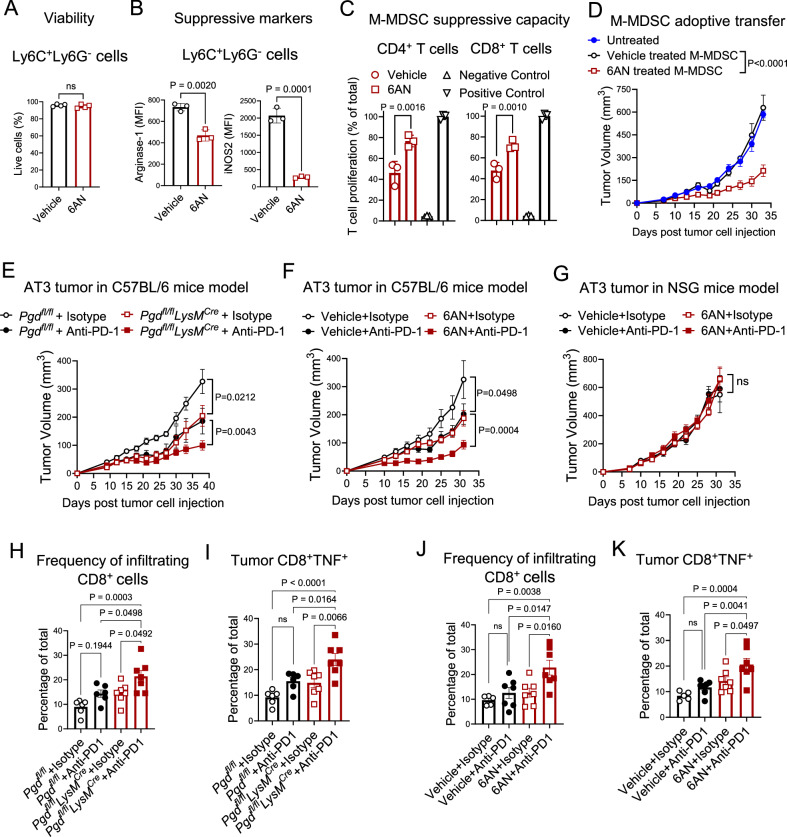
Pharmacological blockade of 6PGD reduces MDSC immunosuppression and enhances anti-PD-1 immune checkpoint blockade efficacy. **A** For pharmacological inhibition of 6PGD, MDSCs were generated in vitro using IL-6 (40 ng/mL) and GM-CSF (40 ng/mL) for 4 days from the bone marrow (BM) of wild type (WT) mice in the presence of 6AN (5 µM) or vehicle (DMSO). 6AN at 5 µM did not induce significant toxicity on M-MDSCs as measured by Annexin V staining. *n* = 4 per group. Two-tailed t-test. **B**, **C** MDSCs were generated as in (**A**), and Arginase 1 and iNOS2 expression were examined in M-MDSC by flowcytometry (**B**). Immunosuppressive function of sorted M-MDSCs was determined by co-cultured with anti-CD3/anti-CD28 activated T cells (CFSE-labeled) at 1:8 (MDSC: T cell) ratio for 72 h. **C** Data are representative of three independent experiments. *n* = 3; One-way ANOVA. **D** To examine suppressive function of M-MDSCs generated as in (**A**), sorted M-MDSC (1 × 10^6^ cells per mouse) were intravenously injected into subcutaneous (s.c.) AT3 mice tumor model (5 × 10^5^ cells per mouse) on day 3 and day 6 post-tumor induction. Mice receiving only AT3 cells served as controls (Untreated). *n* = 6; Two-way ANOVA. **E** Tumor growth in *Pgd*^*fl/fl*^ and *Pgd*^*fl/fl*^*LysM*^*Cre*^ mice injected subcutaneously with AT3 breast cancer cells, followed by 200 μg anti-PD-1 monoclonal antibody or isotype control (2× weekly; intraperitoneally (i.p.)). *n* = 7 mice per group. Two-way ANOVA. **F**, **G** Tumor growth was measured in WT immunocompetent (**F**) or NSG immunodeficient (**G**) mice injected s.c. with AT3 breast cancer cells (5 × 10^5^ cells per mouse) followed by 6AN (1 mg/kg) or vehicle (1% DMSO) i.p., every 3 days and 200 μg anti-PD-1 depleting antibody or isotype control (2× weekly, i.p.). *n* = 7; Two-way ANOVA. **H**, **I** Flowcytometry analysis of the frequency of tumor-infiltrating CD8^+^ T cells (**H**) and their TNF expression (**I**) at day 38 (endpoint) in the AT3 breast cancer model as in E.  *n* = 7; One-way ANOVA. **J**, **K** Flowcytometry analysis of the frequency of tumor-infiltrating CD8^+^ T cells (**J**) and their TNF expression (**K**) at day 32 (endpoint) experimental mice as in (**F**). *n* = 7; One-way ANOVA. All data are shown as mean ± SEM.

The cellular, metabolic, and molecular data have so far shown that 6PGD plays a fundamental role in M-MDSC metabolic fitness in the TME. We next examined the potential translatability of targeting 6PGD to reprogram the TME to become immunostimulatory using the small molecule inhibitor 6AN. First, we generated M-MDSCs from the BM of wild-type mice in the presence of 6AN (5 µM) and confirmed that 6AN treatment did not induce significant changes in M-MDSC viability (Fig. 6A). However, 6AN treatment significantly reduced the expression of Arginase 1 and iNOS2 immunosuppressive markers in M-MDSCs (Fig. 6B), like the *Pgd*-deficient MDSCs (Fig. 2J). 6AN-treated M-MDSCs were also less suppressive on T cell proliferation in co-culture with activated T cells (CFSE-labeled) for both CD4⁺ and CD8⁺ T cells (Fig. 6C).

The immunoregulatory function of M-MDSCs generated in the presence of 6AN was further examined in an adoptive transfer system in vivo. MDSCs were generated in vitro with 6AN treatment and CD11b⁺Ly6C^+^Ly6G^−^ M-MDSCs were sorted. Adoptive transfer (i.v.) of in vitro generated M-MDSCs on days 3 and 6 showed that tumor growth was reduced in 6AN-treated M-MDSCs compared to controls (Fig. 6D). These results recapitulate the effect of 6PGD blockade in *Pgd-*deficient MDSCs, confirming the potential of 6PGD as a checkpoint to reprogram M-MDSC immunosuppressive function in the TME.

Anti-PD-1 therapy is an effective therapy for selected patients with advanced malignancies; however, many patients eventually develop progressive disease due to profound immunosuppression in the TME^38^. M-MDSCs are key regulators of immunosuppression in the TME and help define the outcomes of ongoing tumor treatments, including the efficacy of immune checkpoint blockade therapy^39,40^. Based on these insights, we examined whether 6AN administration could increase the efficacy of anti-PD-1 immunotherapy.

We used the AT3 model to evaluate the M-MDSC inhibition on the efficacy of anti-PD-1 therapy. AT3 was injected into *Pgd*^*fl/fl*^*LysM*^*Cre*^ and *Pgd*^*fl/fl*^ mice. Once tumors became palpable, tumor-bearing mice received anti-PD-1 monoclonal antibody treatment or isotype (controls) (200 µg per mouse, twice a week for 5 weeks). The efficacy of anti-PD-1 was significantly increased in *Pgd*^*fl/fl*^*LysM*^*Cre*^ mice (Fig. 6E). Next, wild-type mice were inoculated with AT3. When tumors were palpable, tumor-bearing mice were treated with 6AN or vehicle alone or in combination with anti-PD-1. Reduced tumor growth was seen in with anti-PD-1 or with 6AN treatment, with a significantly increased effect with anti-PD-1 and 6AN (Fig. 6F). To confirm that the anti-tumor effect of 6AN is dependent on immune response, immunodeficient NSG mice were inoculated with AT3 and treated with 6AN and anti-PD-1. Monitoring tumor growth showed no difference between the treatment groups (Fig. 6G), indicating that the effect of 6AN on tumor control and enhancement of anti-PD-1 efficacy is immune response dependent.

Examination of tumor-infiltrating immune cells in recipients treated with anti-PD-1 showed that 6PGD deficiency in MDSCs or 6AN administration enhanced the frequency of tumor-infiltrating CD8⁺ cells (Fig. 6H, J) and TNF⁺ CD8⁺ T cells (Fig. 6I, K).

These findings indicate that 6PGD acts as a checkpoint for MDSC immunosuppressive function in the TME and that targeting 6PGD with 6AN improves the efficacy of anti-PD-1 immunotherapy.

### 6PGD inhibition reduces the immunosuppressive function of human myeloid suppressor cells

**Fig. 7:**
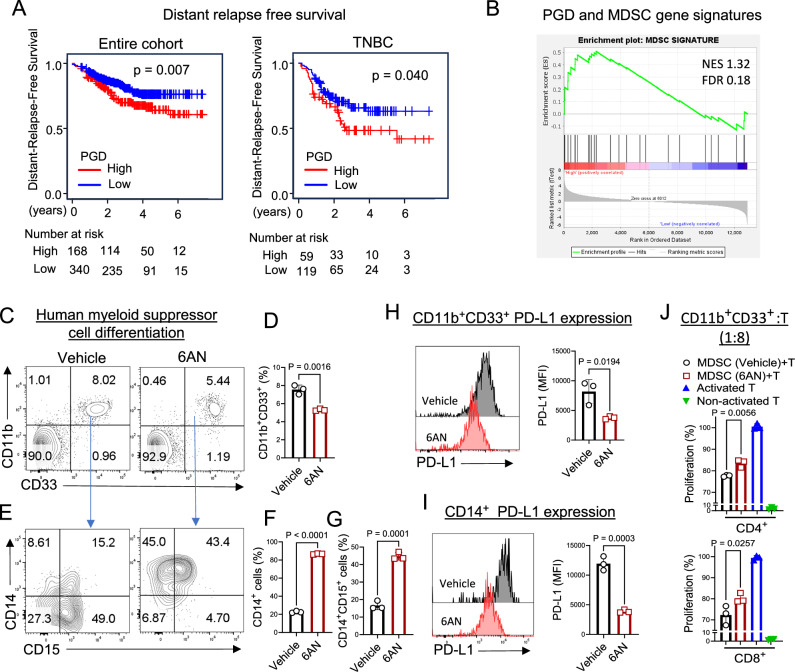
6PGD inhibition decreases the immunosuppressive function of human myeloid suppressor cells; a predictive risk factor for breast cancer outcome. **A** Kaplan‒Meier estimates of distant relapse-free survival in the genomic analysis of breast cancer patients (GSE25066). The impact of *PGD* gene expression in entire cohort and TNBC patient samples are shown. *p* values were calculated using the log-rank test controlled by cohort. Patients were divided into high- and low-PGD expression groups using a higher tertile cutoff. **B** Gene set enrichment analysis (GSEA) of MDSC signatures comparing high and low *PGD* gene expression in GSE25066 cohort. Patients were divided into high- and low-PGD expression groups using a higher tertile cutoff. FDR ˂ 0.25 was considered as significant. **C**–**G** Human myeloid suppressor cells were generated from human peripheral blood mononuclear cells (PBMCs) in vitro using IL-6 (40 ng/mL) and GM-CSF (40 ng/mL) with 6AN (5 µM) or vehicle (DMSO). On Day 6, the frequency of live CD11b^+^CD33^+^ (total MDSC-like cells) (**C**, **D**), CD11b^+^CD33^+^CD14^+^CD15^−^ (monocytic-MDSC-like cells), and CD11b^+^CD33^+^CD14^−^CD15^+^ (PMN-MDSC-like cells) (**E**–**G**) cells were detected by flowcytometry. *n* = 3; Two-tailed t-test. **H**, **I** The expression of PD-L1 suppressive marker in human MDSC like cells (derived as in (**C**–**G**)) with 6AN (5 µM) or vehicle. PD-L1 expression was examined in CD11b^+^CD33^+^ (total MDSC like cells) (**H**) and CD11b^+^CD33^+^CD14^+^ monocytic cells (**I**). Two-tailed t-test. **J** Human MDSC like cells were derived as in (**C**–**G**) with 6AN (5 µM) or DMSO. CD11b^+^CD33^+^ (total MDSC-like cells) were sorted using SONY sorter and co-cultured with anti-CD3/anti-CD28 stimulated CSFE-labeled allogeneic T cells for 72 h. T cell proliferation was evaluated by flowcytometry. *n* = 3; Two-tailed t-test. All data are shown as mean ± SEM.

As shown in Fig. 1A, genes involved in the oxidative branch of the PPP and *PGD* were positively correlated with tumor progression (Fig. 1A). We examined the genomic analysis of breast cancer patients receiving neoadjuvant taxane-anthracycline chemotherapy (GSE25066). Higher *PGD* expression correlated with worse distant relapse-free survival in the entire breast cancer cohort and in a subset of TNBC patients (Fig. 7A). Additionally, GSEA revealed that increased *PGD* gene expression levels were correlated with MDSC gene signatures in the dataset, suggesting high infiltration of MDSC in high *PGD*-expressing tumor (Fig. 7B).

Therefore, we investigated the effect of 6PGD inhibition on human MDSC-like cells. Human myeloid suppressor cells (MDSC-like) were generated in vitro^22,41^ from healthy donor peripheral blood mononuclear cells (PBMCs) in the presence of 6AN (5 µM) or control vehicle. Flowcytometry analysis revealed that 6PGD blockade by 6AN significantly reduced the overall expansion of CD11b^+^CD33^+^ myeloid cells (Fig. 7C, D). Among the differentiated cells, there was a shift towards the development of neutrophil-like cells expressing high levels of CD14^+^CD15^+^ markers and reduced levels of CD14^+^CD15^–^ cells (Fig. 7E–G).

CD14^+^CD15^+^ cells, representing the granulocytic (neutrophil-like) subset of myeloid cells, are associated with lower immunosuppressive activity compared to CD14^+^CD15^−^ (monocytic) cells^5^. Flowcytometry further confirmed that 6AN treatment downregulated the expression of the PD-L1 suppressive marker in both total CD11b^+^CD33^+^ cells (Fig. 7H) and the CD14^+^CD15^-^ monocytic subset (Fig. 7I). In functional assays, sorted CD11b^+^CD33^+^ cells were co-cultured with anti-CD3/anti-CD28 mAb-stimulated autologous T cells (CFSE-labeled). T cell proliferation was higher in the presence of 6AN-treated MDSC-like cells, indicating a reduction in the suppressive capacity of MDSC-like differentiated with 6AN (Fig. 7J).

These findings align with our observations in mouse M-MDSCs, underscoring the critical role of 6PGD in myeloid suppressor cell development and immunosuppressive function. This pathway could be targeted in cancer patients to enhance the effectiveness of immunotherapy, where M-MDSCs are key drivers of immunosuppression in the TME.

## Discussion

Under normal conditions, the PPP is relegated to a relatively minor role in metabolic activity, responsible for less than 10% of glucose utilization^12^. However, in inflammation or conditions of cell-intrinsic or environmental stress, such as in the TME, the PPP becomes activated to drive at least two needs; (1) to produce NADPH for eliminating peroxides (ROS), and (2) to generate deoxyribonucleotides necessary for cell proliferation^42^. In several cell types, including cancer cells^43^, mature neutrophils^44,45^, and T cells^46^, G6PD, the first enzyme of the pathway, is reported to regulate PPP activity. Here, we found that the third enzyme in the PPP, 6PGD, is a critical checkpoint that dictates the phenotype and function of tumor M-MDSCs. Notably, targeting the first enzyme in the pathway, G6PD, had minimal impact on M-MDSCs, consistent with prior findings that G6PD inhibition had limited effects on other myeloid cells, such as macrophages^46^. These findings uncover a key biological pathway in M-MDSCs driven by 6PG, the substrate of the 6PGD enzyme in the PPP, which plays a pivotal role in compromising M-MDSC metabolic fitness. This suggests that 6PGD overexpression in tumor-associated M-MDSCs is a biological response to clear excess 6PG, thereby preserving their metabolic capacity and function. Our results also indicate that M-MDSCs utilize the PPP primarily for nucleotide precursor synthesis. Previous studies in tumor cells support this finding, where 6PGD was found to be upregulated in cancer cells to facilitate DNA synthesis and promote proliferation^47^. However, blocking the 6PGD checkpoint triggers the activation of the non-oxidative PPP branch, sustaining nucleotide precursor biosynthesis as a compensatory mechanism.

The immunosuppressive functions of M-MDSCs in the TME are regulated by several signaling pathways^5,6^. Little is known about the crosstalk between the metabolic pathways and the regulation of intracellular signaling. In tumor cells, the G6PD enzyme product, 6PGL, and the 6PGD enzyme product, Ru5P have been reported to regulate the AMPK mediated signaling^48,49^. Our studies have identified a key role for the 6PG as a protein-binding metabolite, activating the IRS-1-PI3K-AKT signaling axis resulting in M-MDSC metabolic reprogramming. Importantly, we demonstrated that 6PG activates the phosphorylation of IRS-1 at the Ser307 site through JNK1, which controls glucose flux within M-MDSCs. IRS-1 is a key signaling adapter for transferring the signal of insulin receptor (IR)^35^. Induction of IRS-1^S307^ phosphorylation has been identified as a key checkpoint for insulin sensitivity and enhanced glucose consumption in the mice^36^. Phosphorylated IRS-1 activates a cascade of signaling for subsequent function^50^. It is important to note that while we focused on the most differentially affected pathway in WT versus 6PGD^−/−^ M-MDSCs, it is possible that other pathways are also influenced by 6PGD blockade. For instance, JNK1 has been implicated in various pathways^51^, including its role in anti-apoptotic functions in myeloid cells by regulating MCL-1, an anti-apoptotic member of the Bcl-2 family, during oxidative stress-induced apoptosis^52^, as well as promoting M1 macrophage development^53^. Future studies are required to comprehensively identify the signaling pathways affected by 6PGD inhibition in M-MDSCs.

Metabolic fitness is a prerequisite for the immunosuppressive function of MDSCs^8,54^, yet the link between metabolic state and suppressive capacity remains poorly defined. We show that 6PGD blockade elevates mROS levels, which in turn polarizes M-MDSCs toward an M1-like proinflammatory phenotype. This observation aligns with previous studies identifying high mROS as a key driver of M1-like monocyte polarization through MAPK and NF-κB signaling^55,56^. However, further investigation is needed to fully elucidate how elevated mROS levels regulate the M-MDSC immunosuppressive function.

Immune checkpoint inhibitors (ICI) result in clinical benefit for patients with various cancers, although their benefits may be limited to certain patient subsets^57^. Moreover, many ICI-sensitive tumors can develop resistance to ICI. Our preclinical data suggest that targeting the oxidative PPP at the 6PGD checkpoint with small molecule inhibitors like 6AN may help overcome this resistance by reducing the immunosuppressive function of M-MDSCs. The potential clinical relevance of this approach is supported by our finding that 6AN decreases both the generation and immunosuppressive functions of human, MDSC-like populations. It is important to highlight that these studies used peripheral blood from healthy individuals, not cancer patients. Thus, this research serves as proof of concept, demonstrating that 6PGD inhibition can modulate human myeloid cells. There is a need for future studies to achieve a better understanding of 6PGD’s impact on the redox system, to identify potential compensatory pathways for NADPH production, and to evaluate potential clinical toxicity and efficacy. Our results support new research to target 6PGD to enhance the efficacy of ICI immunotherapy. This could offer a significant option for cancer treatment where MDSCs play a pivotal role in disease progression or resistance to treatment.

In summary, we uncovered a key mechanism utilized by tumor-associated M-MDSCs, which involves activating the PPP to prevent the accumulation of 6PG, a metabolite detrimental to their metabolic fitness and immunosuppressive function. Our findings demonstrate that the role of the PPP in TME M-MDSCs extends beyond the standard understanding of its functions, such as NADPH production and nucleotide biosynthesis, underscoring its crucial role in regulating cellular behavior.

## Methods

### Mice and tumor cells

The *C57BL/6J* (B6 CD45.2^+^), *B6.129P2-Lyz2*^*tm1(cre)Ifo*^/J (*LysM*^*Cre*^), and *NOD.Cg-Prkdc*^*scid*^ *Il2rg*^*tm1Wjl*^ *Tg(HLA-A/H2-D/B2M)1Dvs/SzJ* (NSG) mice were purchased from Jackson Laboratories. *Pgd*^*fl/fl*^ mice were provided by Dr. Pankaj Seth (Beth Israel Deaconess Medical Center) and crossed with *LysM*^*Cre*^ mice to generate *Pgd*^*fl/fl*^*LysM*^*Cre*^ mice. Female mice aged 6–12 weeks were age-matched across different groups. All mice were housed in the Roswell Park Comparative Oncology Shared Resources (COSR) facility. They were kept on a 12/12-h light/dark cycle, at 22–26 °C, with an average of 50% humidity, and given sterile pellet food and water *ad libitum*. CO_2_ inhalation was used for murine euthanasia. Animal work was conducted in accordance with the Institutional Animal Care and Use Committee (IACUC)-approved protocol (1143 M), following Roswell Park’s animal care guidelines. The EL-4 T lymphoblast (TIB-39) and B16-F10 melanoma (CRL-6475) cell lines were obtained from the American Type Culture Collection (ATCC). These cells were cultured in DMEM supplemented with 10% FBS, 1% L-glutamine, and 1% Penicillin/Streptomycin. The AT3 cell line was provided by Dr. Scott Abrams’ laboratory (Roswell Park) and cultured under the same conditions, with the addition of 1% non-essential amino acids. All cell lines were tested and authenticated for mycoplasma prior to experimental use.

### Tumor models and treatment

To generate tumor mouse models, 5 × 10^5^ cells/mouse of AT3 breast cancer or EL-4 (ATCC TIB-39), or 2 × 10^5^ B16-F10 melanoma (ATCC CRL-6475) cells were resuspended in 50 µL PBS and injected into the mammary fat pad of the mice. For in vivo 6PGD blockade, 6AN (1 mg/kg) or vehicle (1% DMSO) was administered intraperitoneally (i.p.) every 3 days starting on day 7 post tumor injection. Control mice received vehicle on the same treatment schedule. To obtain tumor M-MDSCs, 5 × 10^5^ AT3 breast tumor cells were injected subcutaneously (s.c.) into the mammary fat pad of the mice, and CD11b^+^Ly6C^+^Ly6G^−^F4/80^−^ M-MDSCs were sorted from the tumors on day 30 post-injection using a SONY MA900 sorter (Fig. S4). CD11b^+^Ly6C^+^Ly6G^−^F4/80^−^ myeloid cells sorted from tumor-free mice served as controls. To deplete Ly6C^+^ MDSCs, AT3 tumor-bearing mice were injected i.p. twice a week with 200 μg anti-Ly6C antibody (clone Monts 1, BioXcell) or an isotype control (clone LTF-2, BioXcell), starting on day 7 post-tumor inoculation. For anti-PD-1 immunotherapy experiments, 200 μg/mouse anti-PD-1 (clone RMP1-14, BioXcell) was injected i.p. twice a week, beginning on day 7 post-tumor injection. Tumor growth in all groups was measured every three days, and tumor volume was calculated as (length × width^2^)/2.

### In vitro MDSC generation

Bone marrow (BM) cells were harvested from the femurs and tibias of *Pgd*^*fl/fl*^*LysM*^*Cre*^ and *Pgd*^*fl/fl*^ mice. Red blood cells (RBCs) were removed using RBC lysis buffer. To differentiate BM cells into M-MDSCs, 2 × 10^6^ cells were cultured in 3 mL of complete RPMI medium supplemented with 10% FBS, 1% L-glutamine, 1% Penicillin/Streptomycin, 40 ng/mL recombinant mouse interleukin-6 (rmIL-6), and 40 ng/mL recombinant mouse granulocyte-macrophage colony-stimulating factor (rmGM-CSF) for 4 days^22^. On day 3 of culture, 1 mL of the medium was replaced with fresh medium containing rmIL-6 and rmGM-CSF (40 ng/mL each). To block 6PGD during MDSC differentiation, 6AN (5 µM) or with vehicle (DMSO) (control) were added on day 0 and again when the medium was refreshed on day 3. For N-acetylcysteine (NAC) treatment, NAC (0.5 mM) was added to differentiation media on day 0. CD11b^+^Ly6C^+^Ly6G^−^ M-MDSCs were sorted by flow cytometry on day 4 for subsequent experiments.

### Human MDSC-like cell generation

Healthy donor human peripheral blood mononuclear cells (PBMCs) were cultured in RPMI complete media supplemented with recombinant human interleukin-6 (rhIL-6, 40 ng/mL) and recombinant human granulocyte-macrophage colony-stimulating factor (rhGM-CSF, 40 ng/mL) for 6 days to generate human myeloid suppressor cells in vitro. On day 3, 1 mL of the media was removed and replaced with 2 mL of fresh media containing rhIL-6 and rhGM-CSF (40 ng/mL each). To block 6PGD, 6AN (5 µM) or vehicle (DMSO) (control) were added on day 0 and included in the media replacement on day 3. Cells harvested on day 6 were examined by flowcytometry for different subpopulations, and total CD11b^+^CD33^+^ myeloid cells were sorted for subsequent analyses.

### Evaluation of M-MDSCs suppressive capacity

The suppressive capacity of MDSCs was examined both in vitro and in vivo.

In vitro evaluation: Mouse M-MDSCs were prepared as described above and co-cultured with plate-bound anti-CD3/anti-CD28-stimulated T cells (CFSE-labeled) at a 1:8 (M-MDSC: T cell) ratio. Proliferation of CD4^+^ and CD8^+^ T cells was assessed by flowcytometry. Controls were unstimulated T cells (negative control) and T cells alone stimulated with anti-CD3 and anti-CD28 (positive control). For human MDSCs, CD11b^+^CD33^+^ MDSCs were sorted and co-cultured with plate-bound anti-CD3-stimulated T cells (CFSE-labeled) at 1:8 (M-MDSC: T cell) ratio. Additionally, soluble anti-CD28 (5 µg/mL) and rhIL-2 (100 ng/mL) were added to the human cell culture. Controls were unstimulated T cells (negative control) and T cells stimulated with plate-bound anti-CD3, soluble anti-CD28 (5 µg/mL), and rhIL-2 (100 ng/mL) (positive control).

In vivo evaluation: To assess the immunosuppressive capacity of 6PGD-blocked M-MDSCs, M-MDSCs were generated from mouse BM as described. CD11b^+^Ly6C^+^Ly6G^−^F4/80^−^ M-MDSCs were adoptively transferred into tumor mouse models. For this, AT3 tumor cells (5 × 10^5^ cells per mouse) were injected s.c. into the mammary fat pad of wild-type mice, and M-MDSCs were adoptively transferred intravenously on days 3 and 6 post tumor inoculation.

### Flowcytometry analysis

To obtain cells from mouse tumors, the following procedure was used: Tumor tissue was harvested and mechanically dissociated, followed by a 30-min treatment with Collagenase/Hyaluronidase at 37 °C. The tissue was then passed through a 70 μm cell strainer, and RBCs were lysed.

For in vitro cultured cells: MDSCs were harvested on day 4 of differentiation. The harvested cells were suspended in FACS buffer (1% FBS in PBS) and stained for surface markers using anti-CD11b-BUV395 (M1/70), Ly6C-BV421 (HK1.4), Ly6G-APC or -PE (1A8), F4/80-PE (BM8), and Aqua LIVE/DEAD Fixable Dead Cell Stain dye at 4 °C for 30 min. For intracellular staining, cells were fixed with fixation buffer (BD Biosciences) according to the manufacturer’s protocol. After 30 min of fixation, cells were permeabilized with fixation/permeabilization buffer (BD Biosciences) and stained with anti-Arg1-FITC (R&D: IC5868F) and iNOS2-PE (W16030C) in permeabilization buffer for 45 min at room temperature (RT).

In M-MDSC suppression assays: co-cultured T cells were surface-stained with anti-CD4-APC (GK1.5), CD8α-PE (53-6.7), and Aqua LIVE/DEAD Fixable Dead Cell Stain dye.

For human myeloid suppressor cells: differentiated cells were harvested on day 6, suspended in FACS buffer, and stained for surface markers using anti-CD11b-APC (ICRF44), CD14-PE (M5E2), CD15-BV785 (W6D3), CD33-PerCP/Cyanine5.5 (WM53), PD-L1-BV711 (29E.2A3), and Aqua LIVE/DEAD Fixable Dead Cell Stain dye for 30 min. Surface-stained cells were then fixed and permeabilized with fixation/permeabilization buffer (BD Biosciences) and stained with anti-Arg1-FITC (R&D: IC5868F) for 45 min at RT (Table S1).

### Annexin V staining

The viability of generated M-MDSCs was assessed using the APC Annexin V Apoptosis Kit (BioLegend). Briefly, generated MDSCs were harvested, suspended in FACS buffer (1% FCS in PBS), and stained for surface markers (CD11b, Ly6C, and Ly6G) as well as Aqua LIVE/DEAD Fixable Dead Cell Stain for 30 min at RT. The cells were then washed once with PBS and twice with 1× binding buffer (BioLegend), and resuspended in 200 µL of 1× binding buffer containing 5 µL of APC Annexin V. After a 15-min incubation at RT, the cells were washed with 500 µL of 1× binding buffer and analyzed using the BD LSRFortessa Cell Analyzer (BD Biosciences).

### Mitochondrial ROS (mROS) assay

To detect levels of mROS generation, M-MDSCs were prepared as described and resuspended in FACS buffer (PBS containing 2% FBS). The cells were incubated with antibodies for MDSC surface markers (CD11b, Ly6C, and Ly6G) along with Aqua LIVE/DEAD Fixable Dead Cell Stain for 30 min at 37 °C. For mROS detection, 5 µM MitoSOX Deep Red (ThermoFisher) was added during the last 10 min of incubation. After washing with FACS buffer, the cells were analyzed using the BD LSRFortessa Cell Analyzer (BD Biosciences) (Table S1).

### Single cell RNA sequencing (scRNA-seq)

To generate AT3 tumor-bearing mice, 5 × 10^5^ AT3 breast cancer cells were injected s.c. into the mammary fat pad of *Pgd*^*fl/fl*^*LysM*^*Cre*^ and *Pgd*^*fl/fl*^ mice (10 mice per group). On day 30, tumors were harvested, digested, and passed through a 70 μm cell strainer. The resulting cell suspensions were pooled, washed, and stained with anti-CD45-PE-Cy7 (30-F11) and Aqua LIVE/DEAD Fixable Dead Cell Stain dye for 30 min at 4 °C. Feature Barcode-conjugated molecules were then bound to cell surface proteins, and live CD45^+^ cells were isolated using the SONY MA900 cell sorter (Sony Biotechnology). Single-cell suspensions of CD45^+^ sorted cells from *Pgd*^*fl/fl*^*LysM*^*Cre*^ and *Pgd*^*fl/fl*^ mouse tumors were pooled and examined using AOPI stain with a Cellometer K2 automated cell counter (Nexcelom) to determine concentration, viability, and to ensure the absence of clumps and debris. Reverse transcription was performed, and amplified cDNA was separated into full-length and Feature Barcode fractions using SPRISelect beads (Beckman Coulter). Full-length amplified cDNA was used to generate libraries, which were evaluated on a D1000 screen tape using a TapeStation 4200 (Agilent Technologies) and quantitated with the Kapa Biosystems qPCR quantitation kit for Illumina. The library was sequenced on a NovaSeq6000 following the manufacturer’s recommended protocol (Illumina Inc.). Bioinformatics analyses were conducted primarily using the Seurat (v5.0.0) R package for scRNA-seq. Two scRNA-seq samples were individually imported into Seurat objects to examine feature number, mitochondrial percentage, and read count distributions within each sample. A total of 2555 cells from the *Pgd*^*fl/fl*^*LysM*^*Cre*^-CD45^+^ sample and 3457 cells from the *Pgd*^*fl/fl*^-CD45^+^ sample were analyzed. Cells with fewer than 500 features, more than 7500 features, or greater than 15% mitochondrial content were filtered out. Principal component analysis was performed to detect and visualize highly variable genes. Annotated Macrophage, Monocyte, and Neutrophil cells were selected as target Myeloid cell populations. Differential gene expression analysis was conducted using Seurat’s FindMarkers function with a Wilcoxon Rank Sum test. Gene-set enrichment analyses (GSEA) were performed on comparisons of interest using the GSEA pre-ranked procedure in the clusterProfiler package (v4.6.2) with the msigDB database from the msigdbr (v7.5.1) package. Mouse versions of Hallmark, C2, and C5-GO gene sets were used for pathway analyses. Negative log *p*-values multiplied by the sign of the avg_log2FC values (from FindMarkers results) were utilized as ranked values for pathway analyses. Specific cell-level signature scores were obtained using Seurat’s AddModuleScore function. Functional differential gene signature densities between *Pgd*^*fl/fl*^*LysM*^*Cre*^ and *Pgd*^*fl/fl*^ were assessed using qusage (v2.32) for antigen presentation, phagocytosis, and inflammatory response gene sets.

### Chromatin immunoprecipitation-quantitative polymerase chain reaction (ChIP-qPCR)

BM cells (12 × 10^6^) were harvested and treated with PBS or IL6 (40 ng/mL) at 37 °C, 5% CO_2_. After 30 min, cells were collected and analyzed using the Pierce Magnetic ChIP kit (ThermoFisher) according to the manufacturer protocol. Cells were crosslinked in 1% formaldehyde at RT for 10 min and neutralized with 1× glycine solution. Cells were washed with PBS with 1× Halt Proteinase Phosphatase inhibitor, then incubated with 200 µL membrane extraction buffer/4 × 10^6^ cells for 10 min on ice. Isolated nuclei were digested in 200 µL/4 × 10^6^ cells of MNase Digestion buffer with 1 mM DTT and 10 U of MNase for 15 min at 37 °C. Digested nuclei were resuspended in 100 µL/4 × 10^6^ cells of IP dilution buffer + 1× Halt Proteinase Phosphatase inhibitor and sonicated using the BioruptorTM UCD-200 water bath sonication device (Diagenode) on the “High” setting for 8 min (30 s ON/OFF cycles). The supernatant containing the fragmented chromatin was equally distributed for immunoprecipitation. 10% of the volume distributed was retained as the input control. Fragmented chromatin was incubated with either anti-pSTAT3 (Tyr705) (clone: D3A7), anti-H3 (clone: D2B12), or normal rabbit IgG (Cell Signaling) antibody at 4 °C for 19 h while mixing. Antibody-bound chromatin was isolated with the provided Protein A/G Magnetic Beads by incubating for 3.5 h at 4 °C with mixing followed by magnetic isolation for 3 min. Magnetic bead-bound chromatin was washed three times with 1× IP dilution buffer and once with 1× IP dilution buffer + 350 mM NaCl by rotating at 4 °C for 5 min and was eluted by heating at 65 °C for 40 min. The eluted chromatin and the input samples were treated with Proteinase K for an additional 1.5 h at 65 °C. The isolated DNA was purified and concentrated using the provided spin columns and stored at −20 °C until used in downstream qPCR analysis. qPCR was performed using the SYBR Green PCR Master Mix (ThermoFisher) with the following cycling conditions: 95 °C for 10 min, 40× cycles of 95 °C for 15 s followed by 60 °C for 1 min. Melt curve was generated with the following conditions: 95 °C for 15 s, 60 °C for 1 min, and continuous measurement until 95 °C. Primers used for qPCR are listed in Fig. S2. Data was analyzed as fold change over input.

### Examination of gene expression by qPCR

Expression of *Pgd* gene was examined by real-time quantitative PCR using the ABI 7300 Real-Time PCR system (Applied Biosystems). Total RNA was extracted with RNeasy Mini Kit (QIAGEN) according to the manufacturer's protocol. Total DNA-free RNA was used for mRNA isolation and library construction. For *Pgd*, specific “Mm01263703_m1” Taq-Man probe and the TaqMan RNA-to-CT 1-Step Kit was used (ThermoFisher). After running the samples, expression of *Pgd* was normalized to expression of the housekeeping gene (18S rRNA) as ΔCT = CT (*Pgd*) – CT (18S rRNA). Then, alteration between groups were calculated as fold change = 2^ΔΔCT^. ΔΔCT is: CT (*Pgd*) – CT (*Pgd* at baseline).

### Lentivirus production and stable knockdown cell lines construction

Lentiviruses carrying shRNA were employed to construct cell lines with stable knockdown endogenous *G6pd*, *Pgls*, and *Pgd*. Briefly, to assemble the lentiviruses, lentiviral vector harboring shRNA (Fig. S3E), psPAX2 packaging plasmid and pMD2.G envelope plasmid was co-transfected into HEK293T cells using FuGENE HD Transfection Reagent (Promega) according to the manufacturer’s instructions. Fresh medium was replaced 24 h post-transfection and lentivirus particle-containing supernatant medium was collected 48 and 72 h post-transfection and combined. To generate stable knockdown cells, target cells were infected with harvested lentivirus containing supernatant for 24 h, followed by selected with 1 μg/mL puromycin. CD11b^+^Ly6C^+^Ly6G^−^ M-MDSCs were sorted by flowcytometry on day 4. Knockdown efficiency was evaluated by Western blot.

### IRS-1^S307A^ site-directed mutation

The IRS-1 sequence containing a N-terminal HA tag was obtained by PCR using mouse IRS-1 cDNA as a template and cloned into the appropriate multiple cloning sites of the expression vector pcDNA 3.1(+) (CUSABIO). IRS-1^S307A^ mutation was induced with point mutation containing primers (Table S1) by overlap extension PCR mutagenesis using pcDNA3.1-HA-IRS-1 as a template. Wild-type pcDNA3.1-HA-IRS-1 was removed by DpnI after PCR. BM cells were harvested and then were transfected with pcDNA3.1-HA-IRS-1 or pcDNA3.1-HA-IRS-1^S307A^ via FuGENE HD Transfection Reagent according to the manufacturer’s instructions. Transfected cells were then induced to differentiate to MDSCs as described above. The CD11b^+^Ly6C + Ly6G^−^ M-MDSCs were harvested and examined by Western blot analysis on day 4 of differentiation.

### Immunofluorescence staining

To examine the mitochondrial status within different experimental groups, CD11b^+^Ly6C^+^Ly6G^−^ M-MDSCs were prepared and sorted as described above and then stained with 25 nM MitoTracker Deep Red FM (ThermoFisher Scientific) in complete culture media for 30 min at 37 °C. Then cells were washed and seeded on the slides by Cytospin (Cytospin3, SHANDON) at 2000 rpm for 2 mins. The cells were fixed with 4% PFA and permeabilized with PGS permeabilization buffer (PBS with 0.1% gelatin, 0.05% BSA, and 0.15% saponin). Nucleus was stained with 1 µg/mL DAPI solution (ThermoFisher Scientific). Cells were examined by High Resolution Confocal Microscopy (Leica TCS SP8).

### Transmission electron microscopy (TEM)

MDSCs were generated in vitro in the presence of 6AN (5 µM) or vehicle (DMSO) as described above for 4 days. The harvested MDSCs were fixed in 4% glutaraldehyde and stained for electron microscopy. In brief, cells were fixed in 2.5% glutaraldehyde, 1.25% paraformaldehyde, and 0.03% picric acid in 0.1 M sodium cacodylate buffer for 2 h at RT. Fixed cells were washed in 0.1 M cacodylate buffer and post-fixed with 1% osmium tetroxide (OsO4) and 1.5% potassium ferrocyanide (KFeCN6) for 1 h. The cell pellet was washed with water and maleate buffer (MB) and incubated in 1% uranyl acetate in MB for 1 h. The cells were washed twice with water and dehydrated in 50, 70, 90, and 100% alcohol. Cells were then incubated in propylene oxide for 1 h and infiltrated in a 1:1 mixture of propylene oxide and TAAB Epon (Marivac Canada Inc., St. Laurent, Canada). The cells were embedded in TAAB Epon and polymerized at 60 °C for 2 days. Ultrathin sections (about 60 nm) were cut, put into a copper grid, stained with lead citrate, and examined in a JEOL 1200EX transmission electron microscope or a Tecnai G2 Spirit BioTWIN.

### Phospho-Protein signal profiling

MDSCs were generated in vitro from *Pgd*^*fl/fl*^*LysM*^*Cre*^ and *Pgd*^*fl/fl*^ mice or from wild-type mice in presence of 6AN (5 µM) or vehicle as described above. On day 4, the cells were pelleted and kept at −80 °C until analysis. Phospho-Protein signal profiling using the Phospho Explorer Antibody Array kit (Full Moon BioSystems, PEX100) was performed according to the manufacturer’s instructions. In short, cells were lysed in the provided extraction buffer with 1× Halt proteinase/phosphatase inhibitor by vortexing with the provided lysis beads. The supernatant was collected and loaded onto the provided buffer exchange column and samples resuspended in labeling buffer. Protein concentration was determined using PierceTM BCA Protein Assay kit (ThermoFisher) and equal amounts of proteins per sample were labeled with biotin. Microarray slides brought to RT prior to blocking with 3% milk in blocking reagent and washed ten times with milliQ water by vigorous shaking. Biotinylated samples were incubated on the microarray slide in 3% milk in the coupling reagent for 2 h at RT. Microarray slides were washed vigorously with the provided wash buffer, followed by miliQ water to remove any unbound proteins. Slides were conjugated with Cy3-streptavidin (Cytiva GEPA43001) in the provided detection reagent for 20 min at RT. Microarray slides were washed vigorously to limit background and dried by centrifugation. Slides were imaged and analyzed by FullMoon Biosystem’s Array Scanning and & Image Analysis service. In short, slides were scanned, and the median signal intensity (MSI) for each dot was extracted from the array image. Average signal intensity of target replicates was calculated and the value for each target was normalized using background and internal controls (beta-actin, GAPDH and negative controls). Samples were compared as fold change (FC) between groups.

### Western blot analysis

For western blot analysis, CD11b^+^Ly6C^+^Ly6G^−^ M-MDSCs were sorted and lysed in RIPA buffer (ThermoFisher Scientific) containing 1% protease and phosphatase inhibitor (ThermoFisher Scientific) according to the manufacturer’s instructions. Target proteins were separated by SDS-PAGE and transferred to a PVDF membrane (Bio-Rad). The PVDF membrane was blocked by EveryBlot Blocking Buffer (Bio-Rad) and then incubated with appropriate primary antibodies (1:1000) and HRP-conjugated secondary antibodies (1:3000) (Table S1).

### Vectra multispectral imaging

To examine the expression of the 6PGD enzyme in human breast cancer, de-identified breast cancer samples from the Roswell Park Pathology Repository were obtained from five TNBC patients who had undergone surgery. Samples were formalin-fixed paraffin-embedded (FFPE) and sectioned at 4 µm thickness and then mounted on charged slides and dried at 65 °C for 2 h. Samples were deparaffinized using the BOND Dewax solution (AR9222, Leica Biosystems) in a BOND RXm Research Stainer (Leica Biosystems). The developed Akoya Biosciences multispectral immunofluorescence (mIF) staining protocol was used. After staining, Spectral DAPI (Akoya Biosciences) was manually applied to the slides. The mIF panel included the following biomarkers: Ab (Anti-PGD, Catalog# NBP1-31589, Novus Biologicals; Anti-CD14, Catalog# ab183322, Abcam; Anti-CD33, Catalog# ab269456, Abcam; Anti-CD68, Catalog# 76437, Cell Signaling; Anti-AE1/AE3, Catalog# M3515, Agilent Dako. The slides were imaged using the PhenoImager HT (AKOYA Biosciences).

### Immunoprecipitation

Target cells were incubated with appropriate primary antibody and mixed by end-to-end rotation for at least 3 h at 4 °C, followed by incubation with either Pierce Protein A Agarose or Pierce Protein A/G Magnetic Beads (ThermoFisher Scientific) for 2 h. The beads were then washed with 1× PBS for three times and collected either by centrifugation or magnetic stand. Samples were eluted with SDS sample buffer for western blotting analysis.

### JNK1 in vitro kinase assay

Endogenous IRS-1 was immunoprecipitated from mouse brain tissue lysates using anti-IRS-1 antibody (Santa Cruz), and endogenous JNK1 was immunoprecipitated from the 4T1 cell line using JNK1 antibody (Cell Signaling Technologies). Immunoprecipitated JNK1 and IRS-1 were mixed and incubated in Kinase Buffer (Cell Signaling Technologies) supplemented with 200 μM ATP (Cell Signaling Technologies) for 30 min at 37 °C in the presence of increasing concentration of 6PG (0–1000 μM). After 30 min the western blot analysis was used to examine the level of IRS-1^S307^ phosphorylation.

### JNK1 and IRS-1 binding assay

Mouse endogenous JNK1 was immunoprecipitated via Pierce Protein A/G Magnetic Beads, and IRS-1 was precipitated via Pierce Protein A Agarose as above. immunoprecipitated JNK1 and IRS-1 were mixed 1:1 ratio together increasing concentration of 6PG (0–1000 μM). The mixture was then incubated for 30 min at RT. After incubation, the magnetic beads were collected by magnetic stand and washed three times with 1× PBS (PH = 7.0). Samples were eluted with SDS loading buffer and examined by western blot for the level of protein binding.

### Bioenergetics analysis by seahorse

In an XFe96 Extracellular Flux Analyzer (Seahorse Bioscience), the Seahorse XF Glycolysis Stress Test Kit (Agilent) was used to analyze the extracellular acidification rate (ECAR; mpH/min). The Seahorse XF Cell Mito Stress Test Kit (Agilent) was used to examine the mitochondrial oxygen consumption rate (OCR; O_2_ pmol/min). For ECAR analyses, sorted CD11b^+^Ly6C^+^Ly6G^−^ M-MDSCs were examined in four stages: basal, glycolysis induction (10 mM glucose), maximal glycolysis induction (2 mM oligomycin), and glycolysis inhibition (100 mM 2-deoxy-glucose (2-DG)). For OCR analyses, CD11b^+^Ly6C^+^Ly6G^−^ M-MDSCs were measured at four consecutive stages: basal respiration, mitochondrial complex V inhibition (+2 mM oligomycin), maximal respiration induction (+1 mM carbonyl cyanide 4-(trifluoromethoxy) phenylhydrazone), and electron transport chain inhibition (+1 mM rotenone and 1 mM antimycin A).

### Stable isotope-resolved metabolomics (SIRM) analysis

To track glucose within the PPP post-6PGD blockade, MDSCs were generated from *Pgd*^*fl/fl*^*LysM*^*Cre*^ and *Pgd*^*fl/fl*^ mice or from wild-type mice in the presence of 6AN (5 µM) or vehicle as described above. On day 4, the harvested MDSCs were incubated with tracing media: Gln/Glc-free RPMI media supplemented with 10% dialyzed FBS (Life Technologies), 20 mM HEPES, 0.05 mM 2-mercaptoethanol, and 1% penicillin-streptomycin, plus 10 mM D7-D-glucose (D7-Glc) (Cambridge Isotope Laboratories). The cells were collected 8 h, washed twice with PBS and once with distilled water, and then quenched with 60% cold acetonitrile in water. Using the solvent partitioning method with a CH_3_CN: H2O (2:1.5:1, v/v) ratio, the polar metabolites were extracted and lyophilized. To detect metabolite concentrations, the polar extracts were measured by Ion Chromatography-Ultra High-Resolution Mass Spectrometry (IC-UHR-MS). For this, lyophilized extracts were analyzed by ion chromatography using a Dionex ICS-5000+ interfaced to a Thermo Fusion Orbitrap Tribrid mass spectrometer (ThermoFisher Scientific). For ion chromatography, an IonPac AS11-HC-4 μm RFIC&HPIC (2 × 250 mm) column and an IonPac AG11-HC-4 μm guard column (2 × 50 mm) were used as previously described^58^. The data was collected as MS1 under UHR conditions. The Thermo TraceFinder (version 3.3) software package was used to determine peak areas, which were normalized for natural abundance. We calculated the concentration of each metabolite, normalized to protein abundance (μmole/g protein)

### Breast cancer patient dataset analyses

Gene expression and clinical data from the publicly available METABRIC cohort (*n* = 1903) were downloaded through cBioportal and GSE25066 and GSE161529 were downloaded through Gene Expression Omnibus (GEO). The oxidative PPP gene signature was defined based on the *G6PD*, *H6PD*, *PGD*, and *PGLS* genes and the score was calculated by gene set variation analysis (GSVA) algorism^59^. Patients were divided into high and low PGD groups using higher tertile cutoff in whole breast cancer and TNBC cohorts. MDSC signature was defined based on their signature genes, which include *CD177, MMP8, DEFA4, MPO, CTSG, MMP9, IRAK3, IL18R1, CST7, CXCL16, IL10RB, LTB4R, TLR5, CD14, MMP25, CKLF, IL4R, NCF4, CSF3R, CTSC, TNF, NFKBIA, TOLLIP, IL17RA, IRAK2, MYD88, IL1B, ARG2, CD84*, and *CTSD*. GSEA was conducted using MDSC signature comparing PGD high and low groups. For human breast cancer scRNA-seq data, within GSE161529, 13 healthy samples and 8 TNBC samples were analyzed, resulting in a total of 126,297 cells and 27,788 genes. Among these cells, 45,195 tumor-infiltrating immune cells (TIICs) were identified and distributed into 16 cell clusters. Myeloid cells were then filtered, resulting in 6262 cells clustered into 17 distinct groups. The expression levels of oxidative PPP checkpoint enzymes were evaluated in TIIC and myeloid cell clusters.

### Statistics and reproducibility

Sample sizes were chosen based on previous publications and were sufficient for statistical analysis. No data was excluded from analysis. Animals were randomly assigned to each group. Randomization was not required for biochemical and in vitro experiments. Cellular and biochemical experiments were not blinded. All statistical analyses were performed using GraphPad Prism 10 (GraphPad) and R software. Differences between groups were analyzed using the Mann–Whitney and Two-tailed t-test (for two groups) and one-way ANOVA (for more than two groups), followed by the post-hoc Tukey test. Results are presented from 3 to 4 experimental repetitions, and in some experiments, such as imaging, the representative results are demonstrated. Two-way ANOVA with Tukey’s post hoc analysis was used to analyze tumor growth. Differences between groups were considered significant at values of *p* < 0.05. In the figures, all data are shown as mean ± SEM with ∗*p* ≤ 0.05, ∗∗*p* ≤ 0.01, ∗∗∗*p* ≤ 0.001, and ∗∗∗∗*p* ≤ 0.0001, ns = not significant.

### Reporting summary

Further information on research design is available in the Nature Portfolio Reporting Summary linked to this article.

## Supplementary information

Supplementary Information

Reporting Summary

Transparent Peer Review file

## Source data

Source data

## Data Availability

The raw and processed sequencing data (single cell RNA-seq) generated in this paper have been deposited in the Gene Expression Omnibus (GEO) database under accession number (GSE278674). All data supporting the findings of this study are available within the article or in the Supplementary Information or from the corresponding author upon reasonable request. Source data are provided with this paper.
