# Supplementary material for: 6-Phosphogluconate dehydrogenase promotes mitochondrial fusion and immune suppression in tumor-associated monocytic suppressor cells
Source: Nat Commun. 2026 Jan 14;17:229. doi: 10.1038/s41467-025-68102-8 (PMC12804680; doi:10.1038/s41467-025-68102-8)
Supplement: Supplementary file 1 — Supplementary Information [file 41467_2025_68102_MOESM1_ESM.pdf]

Supplemental Figures:

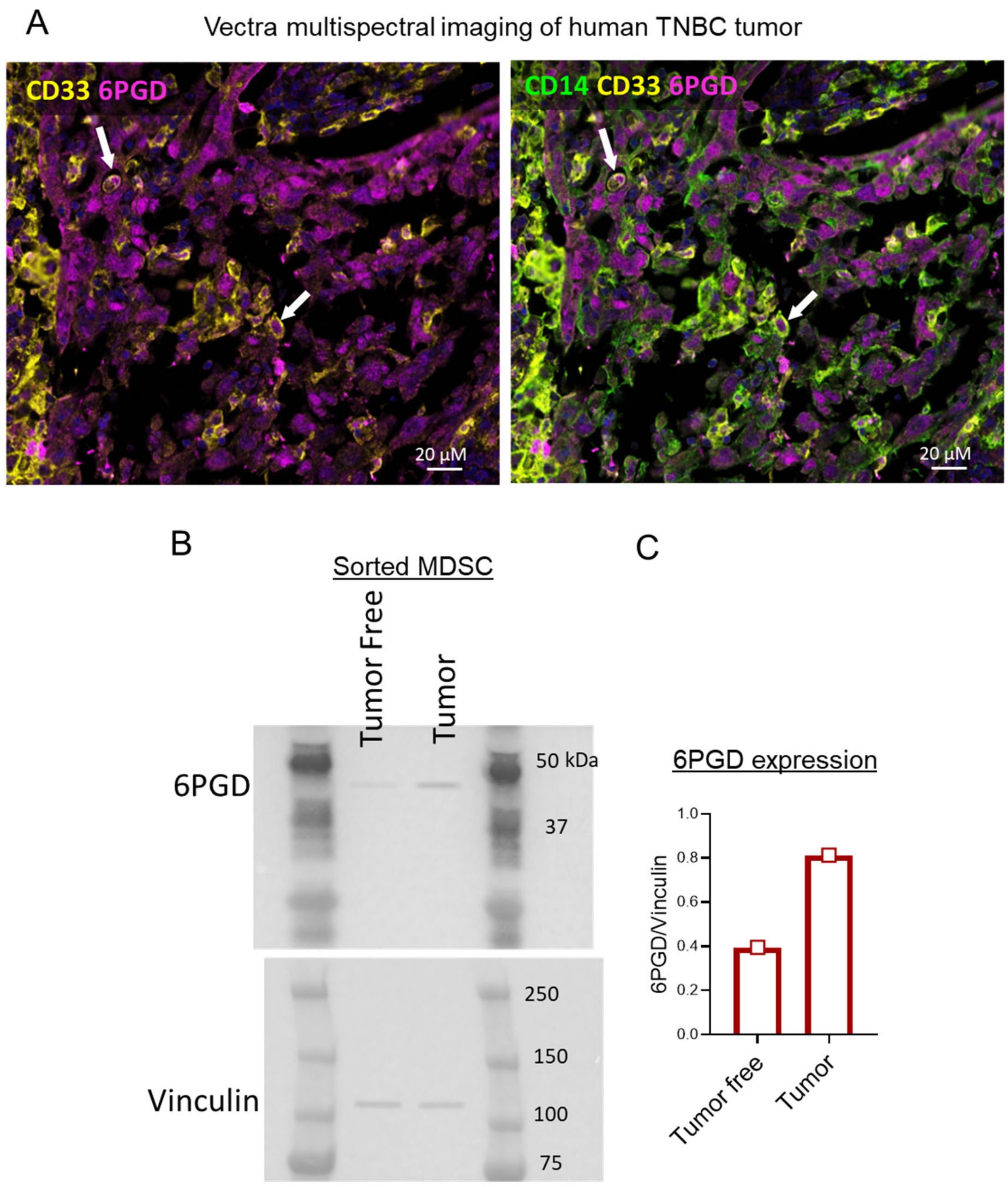

Figure S1: 6PGD expression increases in human and mouse breast cancer M-MDSCs.

(A) Vectra multispectral imaging of a human triple negative breast cancer demonstrates expression of 6PGD (purple) in M-MDSCs stained for CD33 (yellow) and CD14 (green) markers. The arrows point to representative $CD33^{+}CD14^{+}$ (M-MDSC) cells expressing the 6PGD enzyme. Representative of one TNBC tumor.

(B-C) The western blot analysis and relative density quantification of 6PGD expression in mouse MDSCs. AT3 tumor cells (5×10^{5}) were subcutaneously injected into recipient mice. At 35 days post tumor injection, MDSCs were sorted from the tumor of AT3 tumor bearing or BM of tumor free mice using SONY sorter. The expression of 6PGD was assessed by using immunoblot. Vinculin protein serves as the control. Results are representative of two independent experiments with n=1.

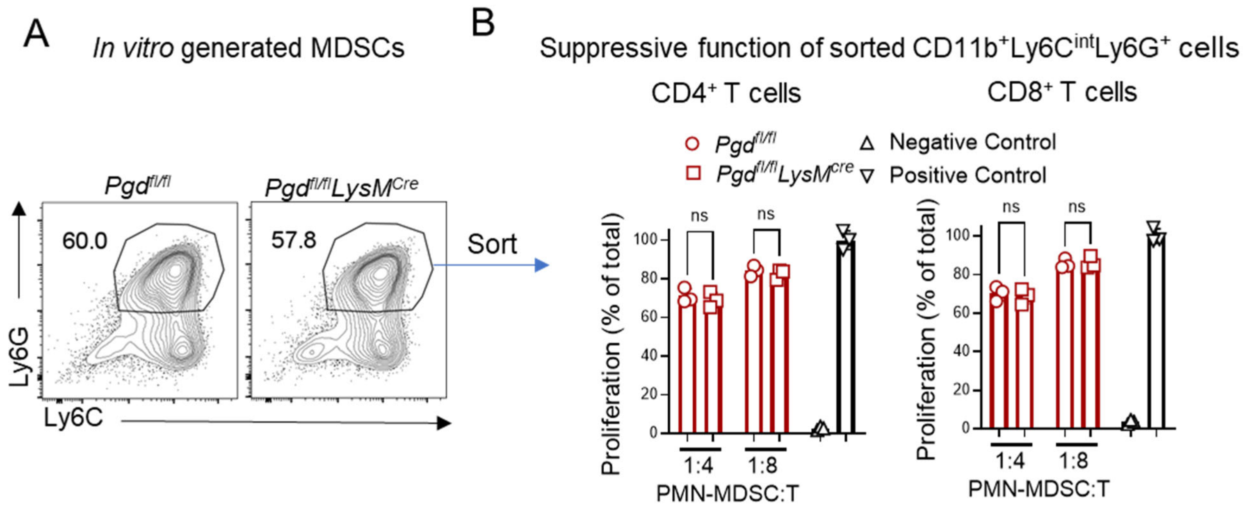

Figure S2: 6PGD deficiency in PMN-MDSCs has minimal effect on their T cell suppressive function.

(A-B) MDSCs were generated *in vitro* from the BM of *Pgd*$^{fl/fl}$ and *Pgd*$^{fl/fl}$*LysM*^{Cre} mice using IL-6 (40 ng/mL) and GM-CSF (40 ng/mL) for 4 days. CD11b^{+}Ly6C^{int}Ly6G^{+} PMN-MDSCs were sorted by flow cytometry. Harvested cells were co-cultured with anti-CD3/anti-CD28 monoclonal antibody (mab)-activated T cells (CFSE-labeled) at 1:4 and 1:8 (MDSC: T cell) ratios. The suppressive capacity of isolated PMN-MDSCs on CD4^{+} and CD8^{+} T cell proliferation was evaluated at 72 hrs. Data are representative of two independent experiments. n=3 per group; One-way ANOVA analyzed statistical significance among groups.

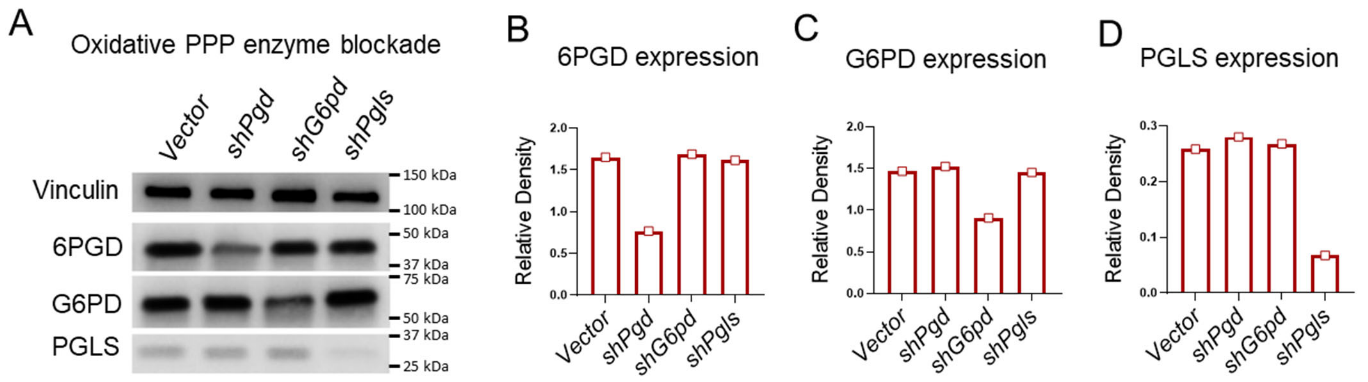

E

shRNA sequences used for stable knockdown of oxidative PPP enzymes

Target	Forward Oligos (5-3)	Reverse Oligos (5-3)	TCR clone ID	Ref Seq	Validated
shPgd-1	TCGAGCGGGATAGAGTATGGAGACATGGA TCCATGTCTCCATACTCTATCCCGTTTTTA	CGCGTAAAAACGGGATAGAGTATGGAGAC ATGGATCCATGTCTCCATACTCTATCCCG	N/A	NM_001081274.2	This study (High efficacy)
shPgd-2	TCGAGCGAGAAATTAGTACCCTTGTTGGAT CCAACAAGGGTACTAATTTCTCGTTTTTA	CGCGTAAAAACGAGAAATTAGTACCCTTGT TGGATCCAACAAGGGTACTAATTTCTCG	N/A	NM_001081274.2	This study (Low efficacy)
shPgd-3	TCGAGCCTCTATGCTTCCAAGATCATGGAT CCATGATCTTGGAAGCATAGAGGTTTTTA	CGCGTAAAAACCTCTATGCTTCCAAGATCA TGGATCCATGATCTTGGAAGCATAGAGG	N/A	NM_001081274.2	This study (Low efficacy)
shG6pd	TCGAGACGTGGTCCTTGGCCAATATGGGAT CCCATATTGGCCAAGGACCACGTTTTTA	CGCGTAAAAAACGTGGTCCTTGGCCAATAT GGGATCCCATATTGGCCAAGGACCACGT	TRCN0000313974	NM_008062.3	Sigma
shPgls	TCGAGCGCAGCCCAAAGTATCATCTTGGAT CCAAGATGATACTTTGGGCTGCGTTTTTA	CGCGTAAAAACGCAGCCCAAAGTATCATCT TGGATCCAAGATGATACTTTGGGCTGCG	TRCN0000101565	NM_025396.3	Sigma

Figure S3: Stable knockdown of oxidative PPP enzymes in M-MDSCs by shRNA.

(A-D) Oxidative PPP checkpoints (6PGD, 6GPD and PGLS) were blocked by short hairpin RNA (shRNA) (*shG6pd*, *shPgd and shPgls*) in MDSCs, generated *in vitro* using IL-6 (40 ng/mL) and GM-CSF (40 ng/mL) for 4 days from the BM of wild type (WT) mice. CD11b^{+}Ly6C^{+}Ly6G^{-} M-MDSCs were sorted by flow cytometry. Expression of 6PGD (A, B), G6PD (A, C) and PGLS (A, D) was detected by western blot analysis on sorted M-MDSC. Vinculin protein served as the control. Results are representative of two independent experiments. n=1 per group.

(E) The table demonstrates the sequence of shRNAs that were tested to block the oxidative PPP enzymes in the CD11b^{+}Ly6C^{+}Ly6G^{-} M-MDSCs. We designed and tested three shRNA targeting *Pgd*. The shPGD-1 showed high efficacy in *Pgd* blockade and was used in subsequent experiments. Mice gene names are presented with standard italicized fonts. The used *shG6pd* and *shPgls* were validated by Sigma Aldrich.

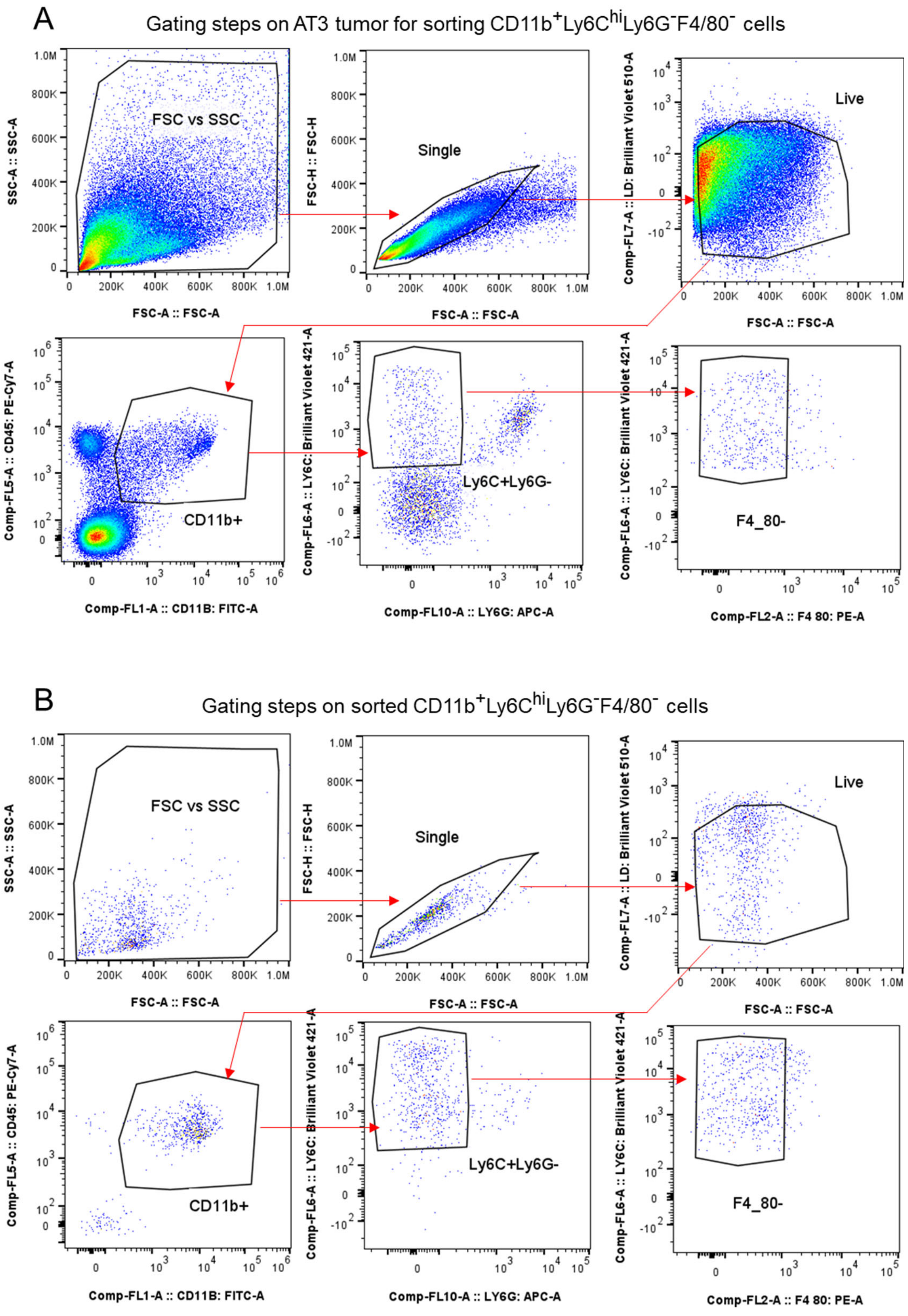

Figure S4: Gating strategy for sorting of CD11b$^+$Ly6C$^+$Ly6G$^-$F4/80$^-$ (M-MDSCs) cells.

(A-B) AT3 tumor cells (5×10^5) were subcutaneously injected into recipient mice. At 35 days post tumor injection, CD11b$^+$Ly6C$^+$Ly6G$^-$F4/80$^-$ (M-MDSCs) were sorted from AT3 tumors using SONY sorter. (B) The purity of sorted cells was checked with the same sorting protocol. Results are representative of two independent experiments.

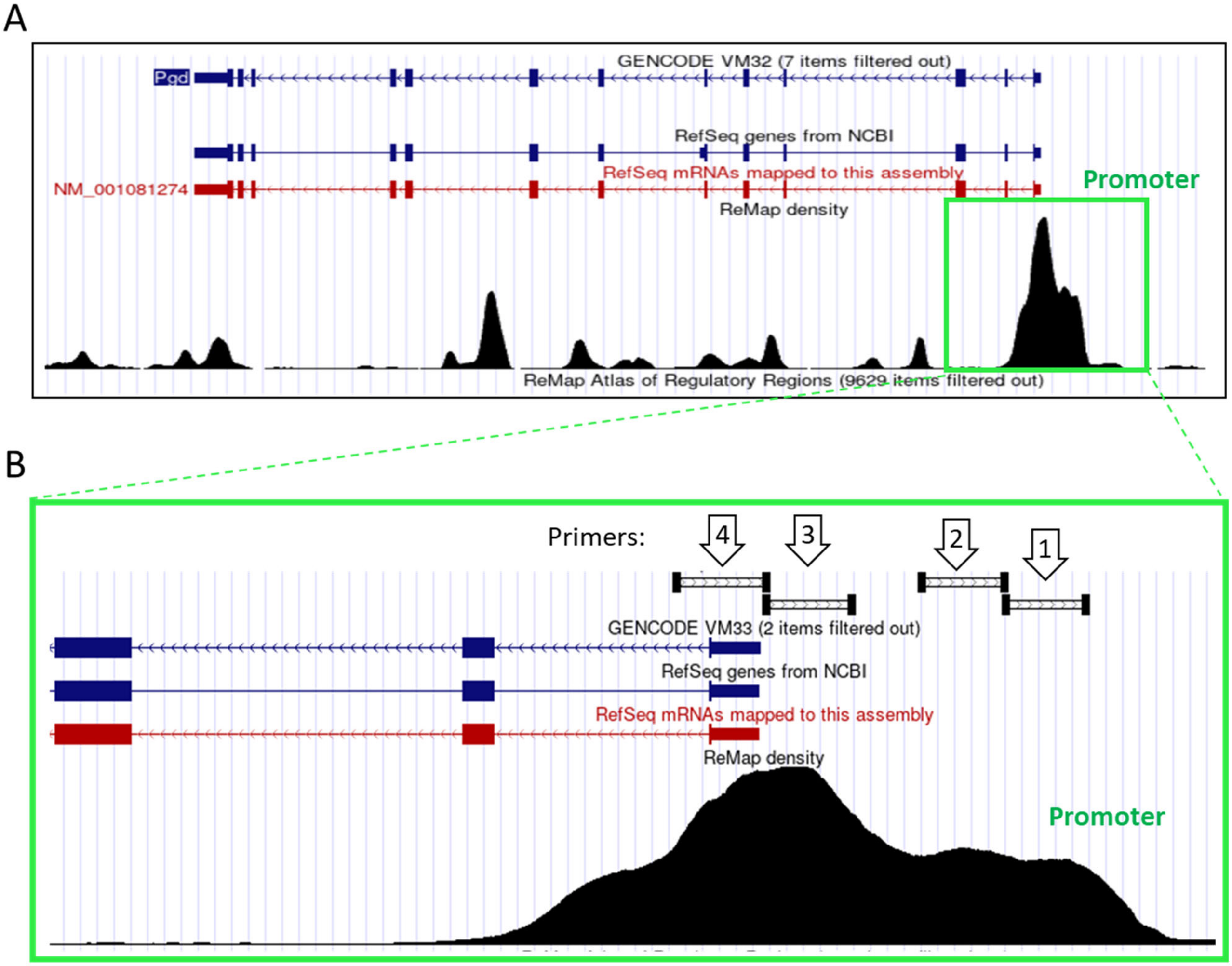

C

		Primer pairs for potential pSTAT3 binding sites
Region #1	Forward:	5'-AGAAAACGAAGCGGAGGAGA-3'
	Reverse:	5'-CTGTAGTGGGGCTGAGGAAA-3'
Region #2	Forward:	5'-GAGGCAGGAGTCAAGTCTGT-3'
	Reverse:	5'-TCCTCCGCTTCGTTTTCTCT-5'
Region #3	Forward:	5'-AATCAGAAGACAGGGGTGGG-3'
	Reverse:	5'-AAGGTCCCGCTAGAACACTT-3'
Region #4	Forward:	5'-AGGGAGACAACTGAGCAGAG-3'
	Reverse:	5'-CCCACCCCTGTCTTCTGATT-3'

Figure S5: ChIP-qPCR primer sets designed for potential pSTAT3 binding sites in the promoter of the *Pgd* gene.

Potential pSTAT3 binding sites in the promoter of mouse *Pgd* are shown in Figure 1M and shown schematically in (A-B). The potential pSTAT3 binding sites are demonstrated according to the UCSC Genome Browser on Mouse (GRCm39/mm39). (B-C) Primer pairs were designed for four potential pSTAT3 binding sites (B), confirmed by Primer 3 software and listed in (C).

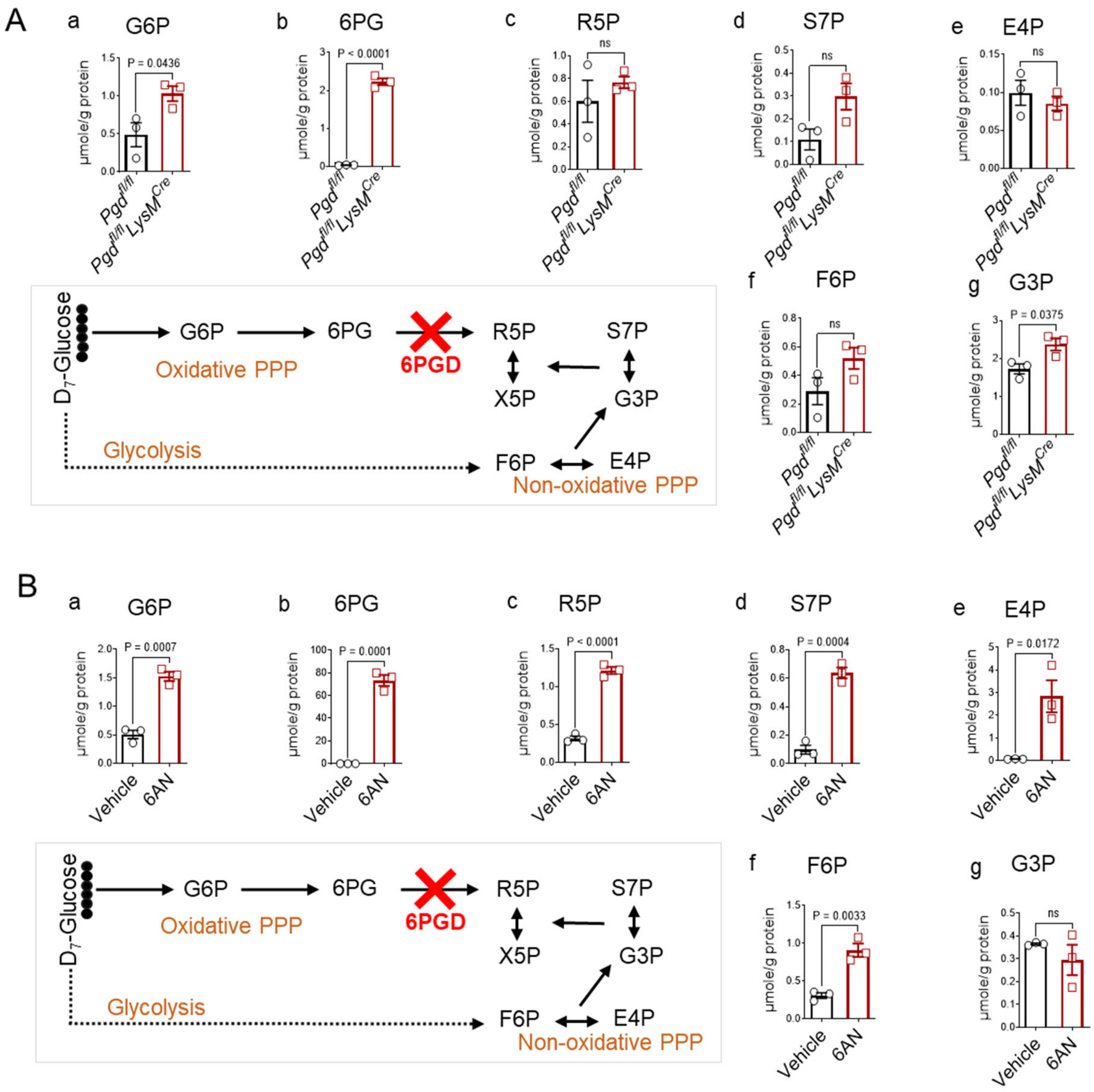

Figure S6: *Pgd$^{fl/fl}$LysM^{Cre}* and 6AN treatment show effective inhibition of oxidative PPP with enhancement of non-oxidative PPP in MDSCs.

(A) MDSCs were generated *in vitro* using IL-6 (40 ng/mL) and GM-CSF (40 ng/mL) from *Pgd$^{fl/fl}$* and *Pgd$^{fl/fl}$LysM^{Cre}* mice bone marrow (BM). On day 4, MDSCs were treated with D_7-glucose. The isotope-labeling patterns of oxidative PPP and non-oxidative PPP metabolites were analyzed at 8 hrs., post treatment by Ion Chromatography-Ultra High-Resolution Mass Spectrometry (IC-UHRMS). Metabolites measured: Glucose-6-Phosphate (G6P), 6-Phosphogluconate (6PG), Ribulose-5-Phosphate (R5P), Sedoheptulose-7-Phosphate (S7P), Erythrose-4-Phosphate (E4P), Glyceraldehyde-3-Phosphate (G3P), Fructose-6-Phosphate (F6P). Xylulose-5-phosphate (X5P) is shown in the figure for context as a metabolite converted to G3P, F6P and R5P. n = 3 per group. Two-tailed *t* test was used to analyze statistical significance between 2 groups.

(B) MDSCs were generated as in A from wild type mice, in the presence of 6AN (5 µM) or vehicle control, and isotope labeled as in A. Incorporation of labeled isotopes into the metabolites of oxidative PPP and non-oxidative PPP metabolites were determined by IC-UHRMS. n = 3 per group. Two-tailed *t* test was used to analyze statistical significance between 2 groups.

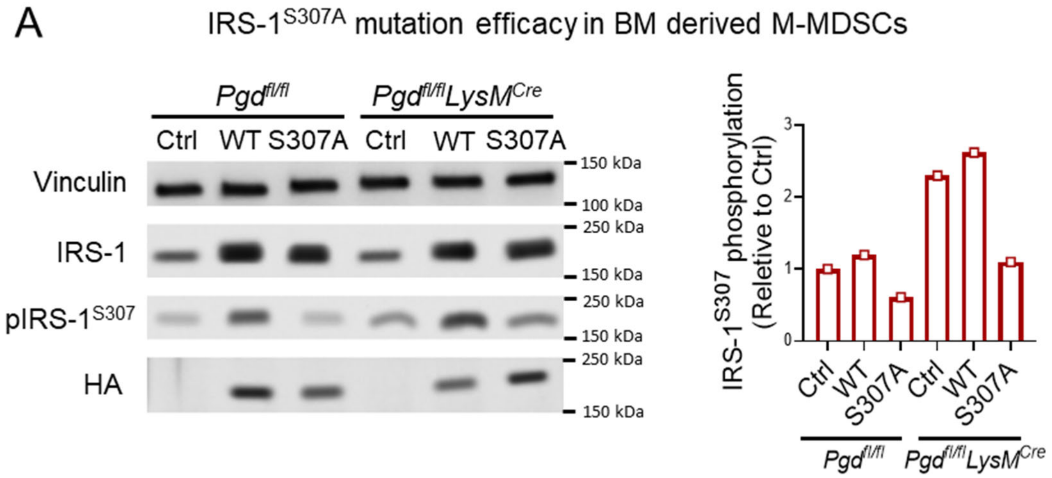

Figure S7: Ser 307 to Ala (S307A) site directed mutation was confirmed in M-MDSCs.

(A) To examine the role of the IRS-1S307 checkpoint in control of M-MDSC immunosuppressive functions, Serine to Alanine (S307A) site specific mutation in IRS-1 was done in $CD11b^{+}Ly6C^{+}Ly6G^{-}$ M-MDSCs. MDSCs were then generated from the bone marrow (BM) of $Pdg^{fl/fl}$ and $Pdg^{fl/fl}LysM^{Cre}$ mice *in vitro* using IL-6 (40 ng/mL) and GM-CSF (40 ng/mL) for 4 days. Transfected $CD11b^{+}Ly6C^{+}Ly6G^{-}$ M-MDSCs were sorted and examined for total IRS1 and $pIRS1^{S307}$ using western blot analysis. Expression of hyaluronic acid (HA) present in the transfecting pcDNA3.1-HA plasmid was used to examine the transfection efficacy. See Materials and Methods for full details regarding the plasmid transfection. Results are representative of two independent repeats with n=1 per group.

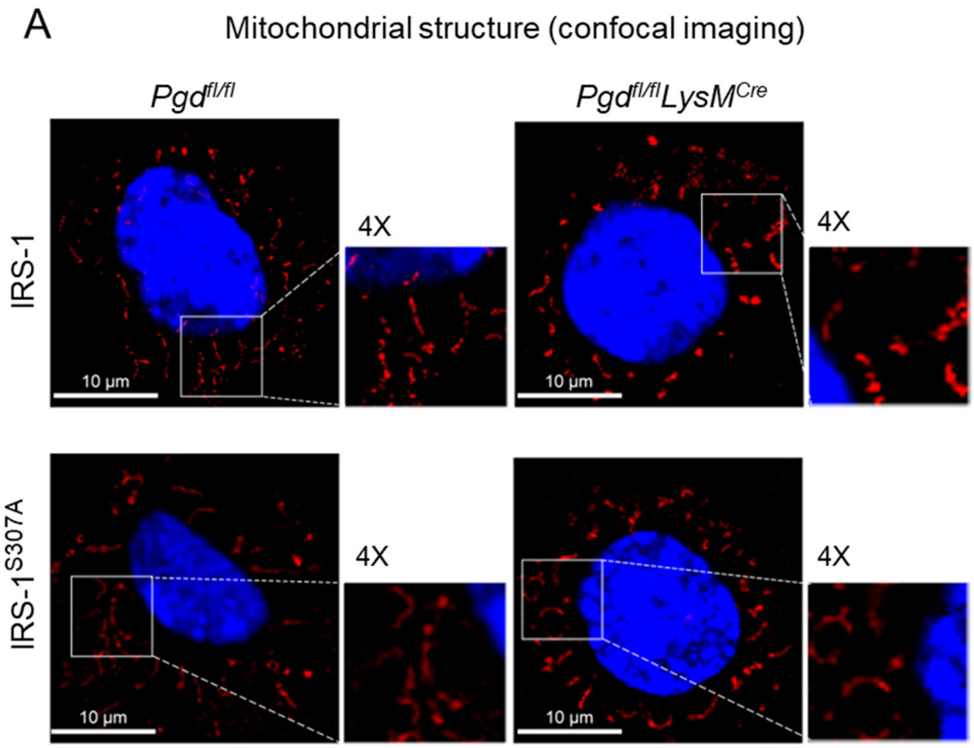

Figure S8: Ser 307 to Ala (S307A) site directed mutation in IRS-1 prevents mitochondrial fission induced by 6PGD blockade in M-MDSCs.

(A) Serine to Alanine (S307A) site specific mutation in IRS-1 or with the or IRS-1S307A control was done in bone marrow (BM) cells from *Pdg*$^{fl/fl}$ and *Pdg*$^{fl/fl}$*LysM*^{Cre} mice. These were differentiated by IL-6 (40 ng/mL) and GM-CSF (40 ng/mL). The M-MDSCs were sorted, and the mitochondrial structures were examined microscopically after staining with MitoTracker Deep Red FM staining and confocal microscopy imaging (as in Figure 4J). The nucleus is stained with DAPI (blue). The mitochondria are shown in red. Result is representative of confocal imaging.

REAGENT or RESOURCE	SOURCE	DILUTION	IDENTIFIER
**Antibodies**			
Anti-mouse/human CD11b Antibody (clone: M1/70) BUV395 conjugated	BD Biosciences	1:400	Cat No# 563553; RRID: AB_2738276
Anti-mouse/human CD11b Antibody (clone: M1/70) PE conjugated	BD Biosciences	1:400	Cat No# 101208; RRID: AB_312791
Anti-mouse Ly-6C Antibody (clone: HK1.4) Brilliant Violet 421 conjugated	BioLegend	1:400	Cat No# 128032; RRID: AB_2562178
Anti-mouse Ly-6G Antibody (clone: 1A8) APC conjugated	BioLegend	1:400	Cat No# 127614; RRID: AB_2227348
Anti-mouse Ly-6G Antibody (clone: 1A8) PE conjugated	BD Biosciences	1:400	Cat No# 551461; RRID: AB_394208
Anti-mouse Nos2 (iNOS) Antibody (clone: W16030C) PE conjugated	BioLegend	1:400	Cat No# 696806; RRID: AB_2876745
Anti-human/mouse Arginase 1/ARG1 Antibody (Polyclonal) FITC conjugated	R&D Systems	1:400	Cat No# IC5868F; RRID: AB_10718118
Anti-mouse CD274 (B7-H1, PD-L1) Antibody (clone: 10F.9G2) Brilliant Violet 711 conjugated	BioLegend	1:400	Cat No# 124319; RRID: AB_2563619
Anti-mouse Gr-1 (Ly-6G/Ly-6C) Antibody (clone: RB6-8C5) PE conjugated	BioLegend	1:400	Cat No# 108406; RRID: AB_313371
Anti-mouse CD4 Antibody (clone: GK1.5) APC conjugated	BioLegend	1:400	Cat No# 100412; RRID: AB_312697
Anti-mouse CD8a Antibody (clone: 30-F11) PE conjugated	BioLegend	1:400	Cat No# 100708; RRID: AB_312747
Anti-mouse CD45 Antibody (clone: 53-6.7) PE-Cy7 conjugated	BioLegend	1:400	Cat No# 103114; RRID: AB_312979
Anti-mouse F4/80 Antibody (clone: BM8) PE conjugated	BioLegend	1:400	Cat No# 123110; RRID: AB_893486
Anti-mouse TNF Antibody (clone: MP6-XT22) PerCP/Cyanine5.5 conjugated	BioLegend	1:400	Cat No# 506322; RRID: AB_961434
Ultra-LEAF Purified anti-mouse CD3ε Antibody (clone: 145-2C11) Unconjugated	BioLegend	-	Cat No# 100340; RRID: AB_11149115
Ultra-LEAF Purified anti-mouse CD28 Antibody (clone: 37.51) Unconjugated	BioLegend	-	Cat No# 102116; RRID: AB_11147170
Anti-human CD11b Antibody (clone: ICRF44) APC conjugated	BioLegend	1:400	Cat No# 301350; RRID: AB_2564134
Anti-human CD14 Antibody (clone: M5E2) PE conjugated	BioLegend	1:400	Cat No# 301850; RRID: AB_2564138
Anti-human CD15 (SSEA-1) Antibody (clone: W6D3) Brilliant Violet 785 conjugated	BioLegend	1:400	Cat No# 323044; RRID: AB_2632921
Anti-human CD33 Antibody (clone: WM53) PerCP/Cyanine5.5 conjugated	BioLegend	1:400	Cat No# 303414; RRID: AB_2074241
Anti-human CD274 (B7-H1, PD-L1) Antibody (clone: 29E.2A3) Brilliant Violet 711 conjugated	BioLegend	1:400	Cat No# 329722; RRID: AB_2565764
Anti-human CD4 Antibody (clone: OKT4) PE/Cyanine7 conjugated	BioLegend	1:400	Cat No# 317414; RRID: AB_571959
Anti-human CD8 Antibody (clone: RPA-T8) Brilliant Violet 421 conjugated	BioLegend	1:400	Cat No# 301036; RRID: AB_10960142
Ultra-LEAF Purified anti-human CD3 Antibody (clone: OKT3) Unconjugated	BioLegend	-	Cat No# 317326; RRID: AB_11150592
Ultra-LEAF Purified anti-human CD28 Antibody (clone: CD28.2) Unconjugated	BioLegend	-	Cat No# 302934; RRID: AB_11148949
Alexa Fluor 647 Donkey anti-rabbit IgG (minimal x-reactivity) Antibody	BioLegend	1:1000	Cat No# 406414; RRID: AB_2563202
Anti-mouse/human PGD Antibody (Polyclonal) Unconjugated	Novus Biologicals	1:1000	Cat No# NBP1-31589; RRID: AB_2299366
Anti-CD33 antibody [EPR23051-101], Unconjugated	Abcam	1:1000	Cat No# ab269456; RRID: AB_2943177

Recombinant Anti-CD14 antibody [SP192], Unconjugated	Abcam	1:1000	Cat No# ab183322; RRID: AB_2909463
CD68 (D4B9C) XP Rabbit mAb, Unconjugated	Cell Signaling Technology	1:1000	Cat No# 76437; RRID: AB_2799882
Monoclonal Mouse Anti-Human Cytokeratin, Clone AE1/AE3, Unconjugated	Agilent Dako	1:1000	Cat No# M3515; RRID: AB_2132885
Histone H3 (D2B12) XP Rabbit mAb (ChIP Formulated) Unconjugated	Cell Signaling Technology	-	Cat No# 4620S; RRID: AB_1904005
Rabbit Anti-STAT3 Antibody XP Rabbit (Polyclonal) Unconjugated	Cell Signaling Technology	1:1000	Cat No# 9132; RRID: AB_331588
Phospho-Stat3 (Tyr705) (clone: D3A7) XP Rabbit mAb Unconjugated	Cell Signaling Technology	1:1000	Cat No# 9145; RRID: AB_2491009
Anti-alpha smooth muscle Actin Antibody (Polyclonal) XP Rabbit Unconjugated	Abcam	1:1000	Cat No# Ab5694; RRID: AB_2223021
Anti-Vinculin (clone: E1E9V) XP Rabbit Unconjugated	Cell Signaling Technology	1:1000	Cat No# 13901 RRID: AB_2728768
Anti-rabbit IgG antibody (polyclonal) Horseradish peroxidase–conjugated	Cell Signaling Technology	1:3000	Cat No# 7074; RRID: AB_2099233
Anti-mouse IgG antibody (polyclonal) Horseradish peroxidase–conjugated	Cell Signaling Technology	1:3000	Cat No# 7076; RRID: AB_330924
Rabbit PGD Polyclonal Antibody Polyclonal Unconjugated	Proteintech	1:1000	Cat No#14718-1-AP RRID: AB_2236801
G6PD Polyclonal Antibody Polyclonal Unconjugated	ThermoFisher Scientific	1:1000	Cat No#A300-404A RRID: AB_2247325
PGLS Polyclonal Antibody Polyclonal Unconjugated	ThermoFisher Scientific	1:1000	Cat No# PA5-31678 RRID: AB_2549151
Tom20 (D8T4N) Rabbit mAb Unconjugated	Cell Signaling Technology	1:1000	Cat No# 42406 RRID: AB_2687663
DRP1 (D6C7) Rabbit MAb Unconjugated	Cell Signaling Technology	1:1000	Cat No# 8570; RRID: AB_10950498
OPA1 (D6U6N) Rabbit mAb Unconjugated	Cell Signaling Technology	1:1000	Cat No# 80471; RRID: AB_2734117
Phospho-DRP1 (Ser616) (D9A1) Rabbit mAb Unconjugated	Cell Signaling Technology	1:1000	Cat No# 4494; RRID: AB_11178659
Phospho-DRP1 (Ser637) (D3A4) Rabbit mAb Unconjugated	Cell Signaling Technology	1:1000	Cat No# 6319; RRID: AB_10971640
MFF (E5W4M) XP Rabbit mAb Unconjugated	Cell Signaling Technology	1:1000	Cat No# 84580 RRID: AB_2728769
Mitofusin-1 (D6E2S) Rabbit mAb Unconjugated	Cell Signaling Technology	1:1000	Cat No# 14739; RRID: AB_ 2744531
Mitofusin-2 (D1E9) Rabbit mAb Unconjugated	Cell Signaling Technology	1:1000	Cat No# 11925; RRID: AB_ 2750893
IRS-1 (D23G12) Rabbit mAb Unconjugated	Cell Signaling Technology	1:1000	Cat No# 3407; RRID: AB_2127860
Phospho-IRS-1 (Ser307) Antibody Polyclonal Unconjugated	Cell Signaling Technology	1:1000	Cat No# 2381; RRID: AB_330342
JNK1 Polyclonal Antibody Polyclonal Unconjugated	ThermoFisher Scientific	1:1000	Cat No# 44-690G; RRID: AB_2533724
PI3 Kinase p85 (19H8) Rabbit mAb Unconjugated	Cell Signaling Technology	1:1000	Cat No# 4257; RRID: AB_659889
Phospho-PI3 Kinase p85 (Tyr458)/p55 (Tyr199) Polyclonal Unconjugated	Cell Signaling Technology	1:1000	Cat No# 4228; RRID: AB_659940
Akt (pan) (C67E7) Rabbit mAb Unconjugated	Cell Signaling Technology	1:1000	Cat No# 4691; RRID: AB_915783
Phospho-Akt (Thr308) (D25E6) XP Rabbit mAb Unconjugated	Cell Signaling Technology	1:1000	Cat No# 13038; RRID: AB_2629447
IRS-1 Antibody (E-12) mouse mAb Unconjugated	Santa Cruz	1:1000	Cat No# sc-8038; RRID: AB_627832

JNK1 Antibody (F-3) mouse mAb Unconjugated	Santa Cruz	1:1000	Cat No# sc-1648; RRID: AB_675868
HA Tag Recombinant Rabbit Monoclonal Antibody (RM305) mAb Unconjugated	ThermoFisher Scientific	1:1000	Cat No# MA5-27915; RRID: AB_2744968
InVivoMAb anti-mouse Ly6C (Clone: Monts1)	BioXcell	-	Cat No# BE0203; RRID: AB_2687696
InVivoMAb rat IgG2b isotype control, anti-keyhole limpet hemocyanin	BioXcell	-	Cat No# BE0090; RRID: AB_1107780
InVivoMAb anti-mouse PD-1 (CD279)	BioXcell	-	Cat No# BE0146; RRID: AB_ 10949053
**Critical commercial assays**			
LIVE/DEAD Fixable Aqua Dead Cell Stain Kit (Aqua)	ThermoFisher		Cat No# L34957
Fixation/Permeabilization Solution Kit with BD GolgiPlug	BD Bioscience		Cat No# 555028
EasySep Mouse T Cell Isolation Kit	STEMCELL Technologies		Cat No# 19851
Pan T Cell Isolation Kit, human	Miltenyi Biotec		Cat No# 130-096-535
EasySep Mouse MDSC (CD11b+Gr1+) Isolation Kit	STEMCELL Technologies		Cat No# 19867
RNeasy Mini Kit	QIAGEN		Cat No# 74104
Pierce BCA Protein Assay Kit	ThermoFisher		Cat No# 23225
Pierce Magnetic ChIP Kit	ThermoFisher		Cat No# 26157
SYBR Green PCR Master Mix	ThermoFisher		Cat No# 4309155
NE-PER Nuclear and Cytoplasmic Extraction Reagents	ThermoFisher		Cat No# 78835
Pierce Protein A/G Magnetic Agarose Beads	ThermoFisher		Cat No# 78610
Phospho Explorer Antibody Array Kit	Full Moon BioSystems		Cat No# PEX100
Mitochondrial Dynamics Antibody Sampler Kit II #74792	Abcam		Cat No# 74792T
APC Annexin V Kit	Biolegends		Cat No# 640920
Seahorse XF Glycolysis Stress Test Kit	Agilent		Cat No# 103020-100
Seahorse XF Cell Mito Stress Test Kit	Agilent		Cat No# 103010-100
**Oligonucleotides**			
Pgd TaqMan Assay probe (FAM-MGB)	ThermoFisher		Cat No# Mm01263703_m1 Assay ID: 4448892
G6pdxTaqMan Assay probe (FAM-MGB)	ThermoFisher		Cat No# Mm04260097_m1 Assay ID: 4448892
Pgls TaqMan Assay probe (FAM-MGB)	ThermoFisher		Cat No# Mm00452601_m1 Assay ID: 4448892
18S rRNA TaqMan Assay probe (VIC-MGB)	ThermoFisher		Cat No#4319413E
**Software and algorithms**			
FlowJo_V10	FlowJo		https://www.flowjo.com/
Graphpad Prism_V9	Graphpad		https://www.graphpad.com/
ImageJ	NIH		https://imagej.net/ij/
TraceFinder_V5	ThermoFisher		https://docs.thermofisher.com/
UCSC Genome Browser on Mouse (GRCm39/mm39)	UCSC		https://genome.ucsc.edu/

Table S1: Information about used antibodies, commercial kits, oligonucleotides and Software.
