# Supplementary material for: 6-Phosphogluconate dehydrogenase promotes mitochondrial fusion and immune suppression in tumor-associated monocytic suppressor cells
Source: Nat Commun. 2026 Jan 14;17:229. doi: 10.1038/s41467-025-68102-8 (PMC12804680; doi:10.1038/s41467-025-68102-8)
Supplement: Supplementary file 2 — Reporting Summary [file 41467_2025_68102_MOESM2_ESM.pdf]

nature portfolio

Corresponding author(s): Hemn Mohammadpour and Saeed Daneshmandi

Last updated by author(s): Nov 11, 2025

Reporting Summary

Nature Portfolio wishes to improve the reproducibility of the work that we publish. This form provides structure for consistency and transparency in reporting. For further information on Nature Portfolio policies, see our Editorial Policies and the Editorial Policy Checklist.

Statistics

For all statistical analyses, confirm that the following items are present in the figure legend, table legend, main text, or Methods section.

n/a	Confirmed	
☐	☒	The exact sample size (n) for each experimental group/condition, given as a discrete number and unit of measurement
☐	☒	A statement on whether measurements were taken from distinct samples or whether the same sample was measured repeatedly
☐	☒	The statistical test(s) used AND whether they are one- or two-sided<br>*Only common tests should be described solely by name; describe more complex techniques in the Methods section.*
☐	☒	A description of all covariates tested
☐	☒	A description of any assumptions or corrections, such as tests of normality and adjustment for multiple comparisons
☐	☒	A full description of the statistical parameters including central tendency (e.g. means) or other basic estimates (e.g. regression coefficient) AND variation (e.g. standard deviation) or associated estimates of uncertainty (e.g. confidence intervals)
☐	☒	For null hypothesis testing, the test statistic (e.g. F, t, r) with confidence intervals, effect sizes, degrees of freedom and P value noted<br>*Give P values as exact values whenever suitable.*
☒	☐	For Bayesian analysis, information on the choice of priors and Markov chain Monte Carlo settings
☒	☐	For hierarchical and complex designs, identification of the appropriate level for tests and full reporting of outcomes
☒	☐	Estimates of effect sizes (e.g. Cohen's d, Pearson's r), indicating how they were calculated

Our web collection on statistics for biologists contains articles on many of the points above.

Software and code

Policy information about availability of computer code

Data collection	TraceFinder_V5 software to collect tracing experiment data
Data analysis	TraceFinder_V5 software to analyze tracing experiment data; Flow cytometry data was analyzed by FlowJo_V10. The results were analyzed by Graphpad Prism_V9 for statistical differences; ImageJ software was used to examine the western blot analysis; UCSC Genome Browser on Mouse (GRCm39/mm39) was used to identify the STAT3 bindings sites.

For manuscripts utilizing custom algorithms or software that are central to the research but not yet described in published literature, software must be made available to editors and reviewers. We strongly encourage code deposition in a community repository (e.g. GitHub). See the Nature Portfolio guidelines for submitting code & software for further information.

Data

Policy information about availability of data

All manuscripts must include a data availability statement. This statement should provide the following information, where applicable:
- Accession codes, unique identifiers, or web links for publicly available datasets
- A description of any restrictions on data availability
- For clinical datasets or third party data, please ensure that the statement adheres to our policy

The scRNA-seq dataset referenced in this paper will be deposited to Gene Expression Omnibus (GEO) database under accession number (GSE278674).

Research involving human participants, their data, or biological material

Policy information about studies with human participants or human data. See also policy information about sex, gender (identity/presentation), and sexual orientation and race, ethnicity and racism.

Reporting on sex and gender	This study does not include human subjects. The breast cancer samples were selected from Roswell Park Comprehensive Cancer Center form a de-identified sample pool independent of sex, gender, race, ethnicity, or other socially relevant characteristics groupings
Reporting on race, ethnicity, or other socially relevant groupings	This study does not include human subjects.
Population characteristics	This study does not include human subjects. Human samples were from patients with breast cancer.
Recruitment	This study does not include human subjects.
Ethics oversight	This study does not include human subjects.

Note that full information on the approval of the study protocol must also be provided in the manuscript.

Field-specific reporting

Please select the one below that is the best fit for your research. If you are not sure, read the appropriate sections before making your selection.

☒ Life sciences ☐ Behavioural & social sciences ☐ Ecological, evolutionary & environmental sciences

For a reference copy of the document with all sections, see nature.com/documents/nr-reporting-summary-flat.pdf

Life sciences study design

All studies must disclose on these points even when the disclosure is negative.

Sample size	The sample size in different experimental groups were determined based on previous studies or preliminary studies.
Data exclusions	Data were not excluded.
Replication	Experiments were done in more that triplicate and each experiment was repeated for two or more repeats to confirm reproducibility.
Randomization	The mice of the in vivo experiments were randomly distributed in different groups before treatments.
Blinding	The data collection and analysis of the results for some of the key experiments including in vitro and in vivo treatments were done by a third investigator (blinding).

Reporting for specific materials, systems and methods

We require information from authors about some types of materials, experimental systems and methods used in many studies. Here, indicate whether each material, system or method listed is relevant to your study. If you are not sure if a list item applies to your research, read the appropriate section before selecting a response.

Materials & experimental systems

n/a	Involved in the study	
☐	☒	Antibodies
☐	☒	Eukaryotic cell lines
☒	☐	Palaeontology and archaeology
☐	☒	Animals and other organisms
☒	☐	Clinical data
☒	☐	Dual use research of concern
☒	☐	Plants

Methods

n/a	Involved in the study	
☒	☐	ChIP-seq
☐	☒	Flow cytometry
☒	☐	MRI-based neuroimaging

Antibodies

Antibodies used	Anti-mouse/human CD11b Antibody (clone: M1/70) BUV395 conjugated; BD Biosciences; Cat No# 563553; RRID: AB_2738276<br>Anti-mouse/human CD11b Antibody (clone: M1/70) PE conjugated; BD Biosciences; Cat No# 101208; RRID: AB_312791<br>Anti-mouse Ly-6C Antibody (clone: HK1.4) Brilliant Violet 421™ conjugated; BioLegend; Cat No# 128032; RRID: AB_2562178

Anti-mouse Ly-6G Antibody (clone: 1A8) APC conjugated; BioLegend ; Cat No# 127614; RRID: AB_2227348
Anti-mouse Ly-6G Antibody (clone: 1A8) PE conjugated; BD Biosciences; Cat No# 551461; RRID: AB_394208
Anti-mouse Nos2 (iNOS) Antibody (clone:W16030C) PE conjugated; BioLegend; Cat No# 696806; RRID: AB_2876745
Anti-human/mouse Arginase 1/ARG1 Antibody (Polyclonal) FITC conjugated; R&D Systems; Cat No# IC5868F; RRID: AB_10718118
Anti-mouse CD274 (B7-H1, PD-L1) Antibody (clone: 10F.9G2) Brilliant Violet 711™ conjugated; BioLegend; Cat No# 124319; RRID: AB_2563619
Anti-mouse Gr-1 (Ly-6G/Ly-6C) Antibody (clone: RB6-8C5) PE conjugated; BioLegend; Cat No# 108406; RRID: AB_313371
Anti-mouse CD4 Antibody (clone: GK1.5) APC conjugated; BioLegend; Cat No# 100412; RRID: AB_312697
Anti-mouse CD8a Antibody (clone: 30-F11) PE conjugated; BioLegend; Cat No# 100708; RRID: AB_312747
Anti-mouse CD45 Antibody (clone: 53-6.7) PE-Cy7 conjugated; BioLegend; Cat No# 103114; RRID: AB_312979
Ultra-LEAF™ Purified anti-mouse CD3ε Antibody (clone: 145-2C11) Unconjugated; BioLegend; Cat No# 100340; RRID: AB_11149115
Ultra-LEAF™ Purified anti-mouse CD28 Antibody (clone: 37.51) Unconjugated; BioLegend; Cat No# 102116; RRID: AB_11147170
Anti-human CD11b Antibody (clone: ICRF44) APC conjugated; BioLegend; Cat No# 301350; RRID: AB_2564134
Anti-human CD14 Antibody (clone: M5E2) PE conjugated; BioLegend; Cat No# 301850; RRID: AB_2564138
Anti-human CD15 (SSEA-1) Antibody (clone: W6D3) Brilliant Violet 785™ conjugated; BioLegend; Cat No# 323044; RRID: AB_2632921
Anti-human CD33 Antibody (clone: WM53) PerCP/Cyanine5.5 conjugated; BioLegend; Cat No# 303414; RRID: AB_2074241
Anti-human CD274 (B7-H1, PD-L1) Antibody (clone: 29E.2A3) Brilliant Violet 711™ conjugated; BioLegend; Cat No# 329722; RRID: AB_2565764
Anti-human CD4 Antibody (clone: OKT4) PE/Cyanine7 conjugated; BioLegend; Cat No# 317414; RRID: AB_571959
Anti-human CD8 Antibody (clone: RPA-T8) Brilliant Violet 421™ conjugated; BioLegend; Cat No# 301036; RRID: AB_10960142
Ultra-LEAF™ Purified anti-human CD3 Antibody (clone: OKT3) Unconjugated; BioLegend; Cat No# 317326; RRID: AB_11150592
Ultra-LEAF™ Purified anti-human CD28 Antibody (clone: CD28.2) Unconjugated; BioLegend; Cat No# 302934; RRID: AB_11148949
Alexa Fluor® 647 Donkey anti-rabbit IgG (minimal x-reactivity) Antibody; Cat No# 406414; RRID: AB_2563202
Anti-mouse/human PGD Antibody (Polyclonal) Unconjugated Novus Biologicals; Cat No# NBP1-31589; RRID: AB_2299366
InVivoMAb anti-mouse PD-1 (CD279) (clone: 29F.1A12™) Unconjugated; BioXCell; Cat No# BE0273; RRID: AB_2687796
InVivoMAb rat IgG2a isotype control, anti-trinitrophenol; BioXCell; Cat No# BE0089; RRID: AB_1107769
Histone H3 (D2B12) XP Rabbit mAb (ChIP Formulated) Unconjugated ; Cell Signaling Technology; Cat No# 4620S; RRID: AB_1904005
Rabbit Anti-STAT3 Antibody XP Rabbit (Polyclonal) Unconjugated; Cell Signaling Technology; Cat No# 9132; RRID: AB_331588
Phospho-Stat3 (Tyr705) (clone: D3A7) XP Rabbit mAb Unconjugated; Cell Signaling Technology; Cat No# 9145; RRID: AB_2491009
Anti-alpha smooth muscle Actin Antibody (Polyclonal) XP Rabbit Unconjugated; Abcam; Cat No# Ab5694; RRID: AB_2223021
Anti-Vinculin (clone: E1E9V) XP Rabbit Unconjugated Cell Signaling Technology; Cat No# 13901; RRID: AB_2728768
Anti-rabbit IgG antibody (polyclonal) Horseradish peroxidase–conjugated ; Cell Signaling Technology; Cat No# 7074; RRID: AB_2099233
Anti-mouse IgG antibody (polyclonal) Horseradish peroxidase–conjugated; Cell Signaling Technology; Cat No# 7076; RRID: AB_330924
Rabbit PGD Polyclonal Antibody Polyclonal Unconjugated; Proteintech; Cat No#14718-1-AP; RRID: AB_2236801
G6PD Polyclonal Antibody Polyclonal Unconjugated; ThermoFisher Scientific; Cat No#A300-404A; RRID: AB_2247325
PGLS Polyclonal Antibody Polyclonal Unconjugated; ThermoFisher Scientific; Cat No# PA5-31678; RRID: AB_2549151
Tom20 (D8T4N) Rabbit mAb Unconjugated; Cell Signaling Technology; Cat No# 42406; RRID: AB_2687663
DRP1 (D6C7) Rabbit MAb Unconjugated; Cell Signaling Technology; Cat No# 8570; RRID: AB_10950498
OPA1 (D6U6N) Rabbit mAb Unconjugated; Cell Signaling Technology; Cat No# 80471; RRID: AB_2734117
Phospho-DRP1 (Ser616) (D9A1) Rabbit mAb Unconjugated; Cell Signaling Technology; Cat No# 4494; RRID: AB_11178659
Phospho-DRP1 (Ser637) (D3A4) Rabbit mAb Unconjugated; Cell Signaling Technology; Cat No# 6319; RRID: AB_10971640
MFF (E5W4M) XP® Rabbit mAb Unconjugated; Cell Signaling Technology; Cat No# 84580; RRID: AB_2728769
Mitofusin-1 (D6E2S) Rabbit mAb Unconjugated; Cell Signaling Technology; Cat No# 14739; RRID: AB_ 2744531
Mitofusin-2 (D1E9) Rabbit mAb Unconjugated; Cell Signaling Technology; Cat No# 11925; RRID: AB_ 2750893
IRS-1 (D23G12) Rabbit mAb Unconjugated; Cell Signaling Technology; Cat No# 3407; RRID: AB_2127860
Phospho-IRS-1 (Ser307) Antibody Polyclonal Unconjugated; Cell Signaling Technology; Cat No# 2381; RRID: AB_330342
JNK1 Polyclonal Antibody Polyclonal Unconjugated; ThermoFisher Scientific; Cat No# 44-690G; RRID: AB_2533724
PI3 Kinase p85 (19H8) Rabbit mAb Unconjugated; Cell Signaling Technology; Cat No# 4257; RRID: AB_659889
Phospho-PI3 Kinase p85 (Tyr458)/p55 (Tyr199) Polyclonal Unconjugated; Cell Signaling Technology; Cat No# 4228; RRID: AB_659940
Akt (pan) (C67E7) Rabbit mAb Unconjugated; Cell Signaling Technology; Cat No# 4691; RRID: AB_915783
Phospho-Akt (Thr308) (D25E6) XP® Rabbit mAb Unconjugated; Cell Signaling Technology; Cat No# 13038; RRID: AB_2629447
IRS-1 Antibody (E-12) mouse mAb Unconjugated; Santa Cruz; Cat No# sc-8038; RRID: AB_627832
JNK1 Antibody (F-3) mouse mAb Unconjugated; Santa Cruz; Cat No# sc-1648; RRID: AB_675868
HA Tag Recombinant Rabbit Monoclonal Antibody (RM305) mAb Unconjugated; ThermoFisher Scientific; Cat No# MA5-27915; RRID: AB_2744968
InVivoMAb anti-mouse Ly6G/Ly6C (Gr-1); BioXcell; Cat No# BE0075; RRID: AB_10312146
InVivoMAb rat IgG2b isotype control, anti-keyhole limpet hemocyanin; BioXcell; Cat No# BE0090; RRID: AB_1107780
InVivoMAb anti-mouse PD-1 (CD279); BioXcell; Cat No# BE0146; RRID: AB_ 10949053

Validation

Anti-mouse/human CD11b Antibody (clone: M1/70) BUV395 conjugated; Reactivity and QC at: RRID: AB_2738276
Anti-mouse/human CD11b Antibody (clone: M1/70) PE conjugated; Reactivity and QC at: RRID: AB_312791
Anti-mouse Ly-6C Antibody (clone: HK1.4) Brilliant Violet 421™ conjugated; Reactivity and QC at: RRID: AB_2562178
Anti-mouse Ly-6G Antibody (clone: 1A8) APC conjugated; Reactivity and QC at: RRID: AB_2227348
Anti-mouse Ly-6G Antibody (clone: 1A8) PE conjugated; Reactivity and QC at: RRID: AB_394208
Anti-mouse Nos2 (iNOS) Antibody (clone:W16030C) PE conjugated; Reactivity and QC at:RRID: AB_2876745
Anti-human/mouse Arginase 1/ARG1 Antibody (Polyclonal) FITC conjugated; Reactivity and QC at: RRID: AB_10718118

Anti-mouse CD274 (B7-H1, PD-L1) Antibody (clone: 10F.9G2) Brilliant Violet 711™ conjugated; Reactivity and QC at: RRID: AB_2563619
Anti-mouse Gr-1 (Ly-6G/Ly-6C) Antibody (clone: RB6-8C5) PE conjugated; Reactivity and QC at: RRID: AB_313371
Anti-mouse CD4 Antibody (clone: GK1.5) APC conjugated; Reactivity and QC at: RRID: AB_312697
Anti-mouse CD8a Antibody (clone: 30-F11) PE conjugated; Reactivity and QC at: RRID: AB_312747
Anti-mouse CD45 Antibody (clone: 53-6.7) PE-Cy7 conjugated; Reactivity and QC at: RRID: AB_312979
Ultra-LEAF™ Purified anti-mouse CD3ε Antibody (clone: 145-2C11) Unconjugated; Reactivity and QC at: RRID: AB_11149115
Ultra-LEAF™ Purified anti-mouse CD28 Antibody (clone: 37.51) Unconjugated; Reactivity and QC at: RRID: AB_11147170
Anti-human CD11b Antibody (clone: ICRF44) APC conjugated; Reactivity and QC at: RRID: AB_2564134
Anti-human CD14 Antibody (clone: M5E2) PE conjugated; Reactivity and QC at: RRID: AB_2564138
Anti-human CD15 (SSEA-1) Antibody (clone: W6D3) Brilliant Violet 785™ conjugated; Reactivity and QC at: RRID: AB_2632921
Anti-human CD33 Antibody (clone: WM53) PerCP/Cyanine5.5 conjugated; Reactivity and QC at: RRID: AB_2074241
Anti-human CD274 (B7-H1, PD-L1) Antibody (clone: 29E.2A3) Brilliant Violet 711™ conjugated; Reactivity and QC at: RRID: AB_2565764
Anti-human CD4 Antibody (clone: OKT4) PE/Cyanine7 conjugated; Reactivity and QC at:RRID: AB_571959
Anti-human CD8 Antibody (clone: RPA-T8) Brilliant Violet 421™ conjugated; Reactivity and QC at: RRID: AB_10960142
Ultra-LEAF™ Purified anti-human CD3 Antibody (clone: OKT3) Unconjugated; Reactivity and QC at: RRID: AB_11150592
Ultra-LEAF™ Purified anti-human CD28 Antibody (clone: CD28.2) Unconjugated; Reactivity and QC at: RRID: AB_11148949
Alexa Fluor® 647 Donkey anti-rabbit IgG (minimal x-reactivity) Antibody; Reactivity and QC at: RRID: AB_2563202
Anti-mouse/human PGD Antibody (Polyclonal) Unconjugated Reactivity and QC at: RRID: AB_2299366
InVivoMAb anti-mouse PD-1 (CD279) (clone: 29F.1A12™) Unconjugated; Reactivity and QC at:RRID: AB_2687796
InVivoMAb rat IgG2a isotype control, anti-trinitrophenol; Reactivity and QC at: RRID: AB_1107769
Histone H3 (D2B12) XP Rabbit mAb (ChIP Formulated) Unconjugated ; Reactivity and QC at: RRID: AB_1904005
Rabbit Anti-STAT3 Antibody XP Rabbit (Polyclonal) Unconjugated; Reactivity and QC at: RRID: AB_331588
Phospho-Stat3 (Tyr705) (clone: D3A7) XP Rabbit mAb Unconjugated; Reactivity and QC at: RRID: AB_2491009
Anti-alpha smooth muscle Actin Antibody (Polyclonal) XP Rabbit Unconjugated; Reactivity and QC at: RRID: AB_2223021
Anti-Vinculin (clone: E1E9V) XP Rabbit Unconjugated Reactivity and QC at: RRID: AB_2728768
Anti-rabbit IgG antibody (polyclonal) Horseradish peroxidase–conjugated ; Reactivity and QC at: RRID: AB_2099233
Anti-mouse IgG antibody (polyclonal) Horseradish peroxidase–conjugated Reactivity and QC at: RRID: AB_330924
Rabbit PGD Polyclonal Antibody Polyclonal Unconjugated; Reactivity and QC at: RRID: AB_2236801
G6PD Polyclonal Antibody Polyclonal Unconjugated; Reactivity and QC at: RRID: AB_2247325
PGLS Polyclonal Antibody Polyclonal Unconjugated; Reactivity and QC at: RRID: AB_2549151
Tom20 (D8T4N) Rabbit mAb Unconjugated; Reactivity and QC at:AB_2687663
DRP1 (D6C7) Rabbit MAb Unconjugated; Reactivity and QC at: RRID: AB_10950498
OPA1 (D6U6N) Rabbit mAb Unconjugated; Reactivity and QC at: RRID: AB_2734117
Phospho-DRP1 (Ser616) (D9A1) Rabbit mAb Unconjugated; Reactivity and QC at: RRID: AB_11178659
Phospho-DRP1 (Ser637) (D3A4) Rabbit mAb Unconjugated; Reactivity and QC at: RRID: AB_10971640
MFF (E5W4M) XP® Rabbit mAb Unconjugated; Reactivity and QC at: RRID: AB_2728769
Mitofusin-1 (D6E2S) Rabbit mAb Unconjugated; Reactivity and QC at: RRID: AB_ 2744531
Mitofusin-2 (D1E9) Rabbit mAb Unconjugated; Reactivity and QC at: RRID: AB_ 2750893
IRS-1 (D23G12) Rabbit mAb Unconjugated; Reactivity and QC at: RRID: AB_2127860
Phospho-IRS-1 (Ser307) Antibody Polyclonal Unconjugated; Reactivity and QC at: RRID: AB_330342
JNK1 Polyclonal Antibody Polyclonal Unconjugated Reactivity and QC at: RRID: AB_2533724
PI3 Kinase p85 (19H8) Rabbit mAb Unconjugated; Reactivity and QC at: RRID: AB_659889
Phospho-PI3 Kinase p85 (Tyr458)/p55 (Tyr199) Polyclonal Unconjugated; Reactivity and QC at: RRID: AB_659940
Akt (pan) (C67E7) Rabbit mAb Unconjugated; Reactivity and QC at: RRID: AB_915783
Phospho-Akt (Thr308) (D25E6) XP® Rabbit mAb Unconjugated; Reactivity and QC at: RRID: AB_2629447
IRS-1 Antibody (E-12) mouse mAb Unconjugated; Reactivity and QC at: RRID: AB_627832
JNK1 Antibody (F-3) mouse mAb Unconjugated; Reactivity and QC at: RRID: AB_675868
HA Tag Recombinant Rabbit Monoclonal Antibody (RM305) mAb Unconjugated; Reactivity and QC at: RRID: AB_2744968
InVivoMAb anti-mouse Ly6G/Ly6C (Gr-1); Reactivity and QC at: RRID: AB_10312146
InVivoMAb rat IgG2b isotype control, anti-keyhole limpet hemocyanin; Reactivity and QC at: RRID: AB_1107780
InVivoMAb anti-mouse PD-1 (CD279); Reactivity and QC at: RRID: AB_ 10949053

Eukaryotic cell lines

Policy information about cell lines and Sex and Gender in Research

Cell line source(s)	AT3 cell line; AT-3 Mouse Breast Tumor Cell Line. AT-3, derived from cells of the primary mammary gland carcinoma of the female MTAG model. The MTAG mouse model was developed using the mouse mammary tumor virus (MMTV) long terminal repeat (LTR) promoter to specifically target the polyomavirus middle T antigen (Ag) expression in the mammary gland tissue of B6 mice.<br><br>EL4 cell line; ATCC; ATCC® TIB-39. EL4 is a T lymphoblast that was established from a lymphoma induced in a male C57BL mouse by 9,10-dimethyl-1,2-benzanthracene. This cell line can be used in immunology research.<br><br>B16F10 cell line; ATCC; ATCC® CRL-6475. The B16 murine melanoma cell line originated in 1954. The tumor spontaneously arose in a C57BL/6J male mouse at the Jackson Laboratories in Maine.

Authentication	AT3 cell line was obtained from Dr. Scott Abrams laboratory and authentication was done in the laboratory. The EL4 and B16F10 cell lines were purchased from ATCC and the authentication was done by ATCC.
Mycoplasma contamination	The cell lines were tested negative for Mycoplasma contamination.
Commonly misidentified lines (See ICLAC register)	There was no misidentified cell line in this study.

Animals and other research organisms

Policy information about studies involving animals; ARRIVE guidelines recommended for reporting animal research, and Sex and Gender in Research

Laboratory animals	C57BL/6J (B6 CD45.2+) from The Jackson Laboratory with Stock No: 000664. Sex: Female; Age: 6-10 weeks old.<br>B6.129P2-Lyz2tm1(cre)Ifo/J (LysMCre) from The Jackson Laboratory with Stock No: : 004781. Sex: Female; Age: 6-10 weeks old.<br>NOD.Cg-Prkdcscid Il2rgtm1Wjl Tg(HLA-A/H2-D/B2M)1Dvs/SzJ (NSG-HLA-A2/HHD) from The Jackson Laboratory with Stock No: 014570. Sex: Female; Age: 6-10 weeks old.<br>Pgdfl/flLysMCre; Sex: Female; Age: 6-10 weeks old.
Wild animals	This study did not involve wild animals.
Reporting on sex	The sex was reported for each experiments. Experiments were done on female mice.
Field-collected samples	This study did not involve field-collected samples.
Ethics oversight	Animal work was done in accordance with the Institutional Animal Care and Use Committee (IACUC)-approved protocol according to Roswell Park animal care guidelines (protocol #1143M).

Note that full information on the approval of the study protocol must also be provided in the manuscript.

Plants

Seed stocks	*Report on the source of all seed stocks or other plant material used. If applicable, state the seed stock centre and catalogue number. If plant specimens were collected from the field, describe the collection location, date and sampling procedures.*
Novel plant genotypes	*Describe the methods by which all novel plant genotypes were produced. This includes those generated by transgenic approaches, gene editing, chemical/radiation-based mutagenesis and hybridization. For transgenic lines, describe the transformation method, the number of independent lines analyzed and the generation upon which experiments were performed. For gene-edited lines, describe the editor used, the endogenous sequence targeted for editing, the targeting guide RNA sequence (if applicable) and how the editor was applied.*
Authentication	*Describe any authentication procedures for each seed stock used or novel genotype generated. Describe any experiments used to assess the effect of a mutation and, where applicable, how potential secondary effects (e.g. second site T-DNA insertions, mosiacism, off-target gene editing) were examined.*

Flow Cytometry

Plots

Confirm that:

- [x] The axis labels state the marker and fluorochrome used (e.g. CD4-FITC).
- [x] The axis scales are clearly visible. Include numbers along axes only for bottom left plot of group (a 'group' is an analysis of identical markers).
- [x] All plots are contour plots with outliers or pseudocolor plots.
- [x] A numerical value for number of cells or percentage (with statistics) is provided.

Methodology

Sample preparation	To obtain cells from mouse tumors, the following procedure was used: Tumor tissue was harvested and mechanically dissociated, followed by a 30-minute treatment with Collagenase/Hyaluronidase at 37°C. The tissue was then passed through a 70 μm cell strainer, and RBCs were lysed. For in vitro cultured cells: MDSCs were harvested on day 4 of differentiation. The harvested cells were suspended in FACS buffer (1% FBS in PBS) and stained
Instrument	Cells were analyzed using the BD LSRFortessa™ Cell Analyzer (BD Bioscience)
Software	Cells were analyzed using FlowJo V10 software.
Cell population abundance	The purity of in vitro generated MDSCs were more that 90% (CD11b+).

Gating strategy

M-MDSC: FSC/SSC, Single cell on FSC/FSH, SSC on Aqua- for Live cell, then Aqua-CD11b+, then Ly6C+Ly6G+
PMN-MDSC: FSC/SSC, Single cell on FSC/FSH, SSC on Aqua- for Live cell, then Aqua-CD11b+, then Ly6C-Ly6G+
CD11b+Gr1+ MDSC: FSC/SSC, Single cell on FSC/FSH, SSC on Aqua- for Live cell, then Aqua-CD11b+, then Gr1+
M-MDSC (Annexin V+): FSC/SSC, Single cell on FSC/FSH, SSC/CD11b+, Ly6C+Ly6G-, then Aqua+Annexin V+
PMN-MDSC (Annexin V+): FSC/SSC, Single cell on FSC/FSH, SSC/CD11b+, Ly6C-Ly6G+, then Aqua+Annexin V+
M-MDSC (Arg1+): FSC/SSC, Single cell on FSC/FSH, SSC on Aqua- for Live cell, then Aqua-CD11b+, then Ly6C+Ly6G-Arg1+
PMN-MDSC (Arg1+): FSC/SSC, Single cell on FSC/FSH, SSC on Aqua- for Live cell, then Aqua-CD11b+, then Ly6C-Ly6G+Arg1+
M-MDSC (iNOS2+): FSC/SSC, Single cell on FSC/FSH, SSC on Aqua- for Live cell, then Aqua-CD11b+, then Ly6C+Ly6G-iNOS2+
PMN-MDSC (iNOS2+): FSC/SSC, Single cell on FSC/FSH, SSC on Aqua- for Live cell, then Aqua-CD11b+, then Ly6C-Ly6G+iNOS2+
CD11b+Gr1+ MDSC (2NBDG+): FSC/SSC, Single cell on FSC/FSH, SSC on Aqua- for Live cell, then Aqua-CD11b+, then Gr1+ 2NBDG+
CD11b+Gr1+ MDSC (MitoTracker+): FSC/SSC, Single cell on FSC/FSH, SSC on Aqua- for Live cell, then Aqua-CD11b+, then Gr1+ MitoTracker +
T cell proliferation: FSC/SSC, Single cell on FSC/FSH, SSC on Aqua- for Live cell, then Aqua-CD4+ or CD8+, then CFSE dilution in each of CD4+ or CD8+
Human MDSC: FSC/SSC, Single cell on FSC/FSH, SSC on Aqua- for Live cell, then Aqua-CD11b+ and CD33+, then examining CD14+ and CD15+ cells
PMN-MDSC (mROS+): FSC/SSC, Single cell on FSC/FSH, SSC on Aqua- for Live cell, then Aqua-CD11b+, then Ly6C-Ly6G+mROS

☒ Tick this box to confirm that a figure exemplifying the gating strategy is provided in the Supplementary Information.
