# Supplementary material for: 6-Phosphogluconate dehydrogenase promotes mitochondrial fusion and immune suppression in tumor-associated monocytic suppressor cells
Source: Nat Commun. 2026 Jan 14;17:229. doi: 10.1038/s41467-025-68102-8 (PMC12804680; doi:10.1038/s41467-025-68102-8)
Supplement: Supplementary file 3 — Transparent Peer Review file [file 41467_2025_68102_MOESM3_ESM.pdf]

nature portfolio

Peer Review File

6-Phosphogluconate dehydrogenase promotes mitochondrial fusion and immune suppression in tumor-associated monocytic suppressor cells

Corresponding Author: Dr Hemn Mohammadpour

This manuscript has been previously reviewed at another journal. This document only contains information relating to versions considered at Nature Communications.

This file contains all reviewer reports in order by version, followed by all author rebuttals in order by version.

Version 0:

Reviewer comments:

Reviewer #1

(Remarks to the Author)
Authors addressed all my questions and concerns with additional experiments and convincing reply. I don't have any more concerns or questions for this manuscript.

Reviewer #3

(Remarks to the Author)
I have no further comments

Reviewer #4

(Remarks to the Author)
In my opinion, the authors have satisfactorily addressed reviewer comments.

Open Access This Peer Review File is licensed under a Creative Commons Attribution 4.0 International License, which permits use, sharing, adaptation, distribution and reproduction in any medium or format, as long as you give appropriate credit to the original author(s) and the source, provide a link to the Creative Commons license, and indicate if changes were made.
In cases where reviewers are anonymous, credit should be given to 'Anonymous Referee' and the source.
The images or other third party material in this Peer Review File are included in the article's Creative Commons license, unless indicated otherwise in a credit line to the material. If material is not included in the article's Creative Commons license and your intended use is not permitted by statutory regulation or exceeds the permitted use, you will need to obtain permission directly from the copyright holder.
To view a copy of this license, visit https://creativecommons.org/licenses/by/4.0/

REVIEWERS' COMMENTS

Reviewer #1 (Remarks to the Author):

Authors addressed all my questions and concerns with additional experiments and convincing reply. I don't have any more concerns or questions for this manuscript.

Response: Thank you.

Reviewer #3 (Remarks to the Author):

I have no further comments

Response: Thank you

Reviewer #4 (Remarks to the Author):

In my opinion, the authors have satisfactorily addressed reviewer comments.

Response: Thank you
